# Therapeutic Potential of *Fridericia platyphylla* (Cham.) L.G. Lohmann: An Integrative Review of the Effects of Crude Extracts and Isolated Phytochemicals in Different Experimental Models

**DOI:** 10.1021/acsomega.5c02027

**Published:** 2025-08-27

**Authors:** Andressa Coelho Ferreira, Raphael Furtado Marques, Icaro Rodrigo Dutra Cunha, Jhonata Costa Moura, Rebekha Matos Oliveira, Kellen De Jesus Farias Da Luz, Ludmila Tavares dos Santos Silva, Mateus Balbino Barbosa de Carvalho, Lara Possapp Andrade, Júlia Karla de Albuquerque Melo Xavier, Joel Felix Silva Diniz Filho, Cláudia Quintino Rocha, Rachel Melo Ribeiro

**Affiliations:** † Biotechnology Graduate Program—RENORBIO, 37892 Federal University of Maranhão, São Luís 65080-805, Brazil; ‡ Health Science Graduate Program, Federal University of Maranhão, Campus São Luís, São Luís 65080-805, Brazil; § Research and Graduate Laboratory in Pharmacology, Federal University of Maranhão, Campus São Luís, São Luís 65080-805, Brazil; ∥ Chemical Graduate Program, Federal University of Maranhão, Campus São Luís, São Luís 65080-805, Brazil; ⊥ Coordination of the Bachelor’s Degree In Natural Sciences—Physics/CCBA, Federal University of Maranhão, Campus Bacabal, Bacabal 65700-000, Brazil

## Abstract

*Fridericia platyphylla* (Cham.) L.G. Lohmann is a species endemic to the Brazilian Cerrado, commonly known as “cervejinha do campo,” “cipó-una,” or ‘tintureiro,” traditionally used to treat kidney stones and joint pain. This species has garnered scientific interest due to its potential pharmacological properties. This review evaluated the pharmacological effects of crude extracts and isolated compounds from *F. platyphylla*, as well as its ethnopharmacological relevance. A comprehensive literature search was conducted across PubMed, Scielo, and Google Scholar for studies published between October 2014 and December 2024, using the descriptors “*F. platyphylla*” and “*A. brachypoda*” combined with terms related to *in vivo*, *in vitro*, and ethnopharmacological research. Of 896 records, 20 studies met the inclusion criteria. The included studies conducted comprehensive isolation and structural elucidation of the bioactive compounds, confirming their chemical identities and supporting their pharmacological relevance through robust analytical and spectroscopic validation. Different extracts and isolated compounds from the roots, leaves, and flowers of *F. platyphylla* have antiulcerogenic, antitumor, antiproliferative, anti-inflammatory, and antifungal action. Brachydins demonstrated cytotoxicity against prostate cancer cells and exhibited biological potential against intracellular amastigotes of *Trypanosoma cruzi*. Luteolin reduced proliferation in U-251 glioblastoma cells with low toxicity to nontumor cells. Additionally, the microemulsion and encapsulation of the active fraction obtained from the roots of this plant showed relevant biological activity for pharmaceutical applications. However, despite these promising findings, potential mutagenic effects raise concerns about the safety of using the plant. In conclusion, *F. platyphylla* and its phytochemicals hold significant therapeutic and technological potential. Nevertheless, further *in vivo* studies are necessary to ensure safety and better understand its pharmacological mechanisms, thereby paving the way for the development of novel therapeutic agents derived from this species.

## Graphical abstract

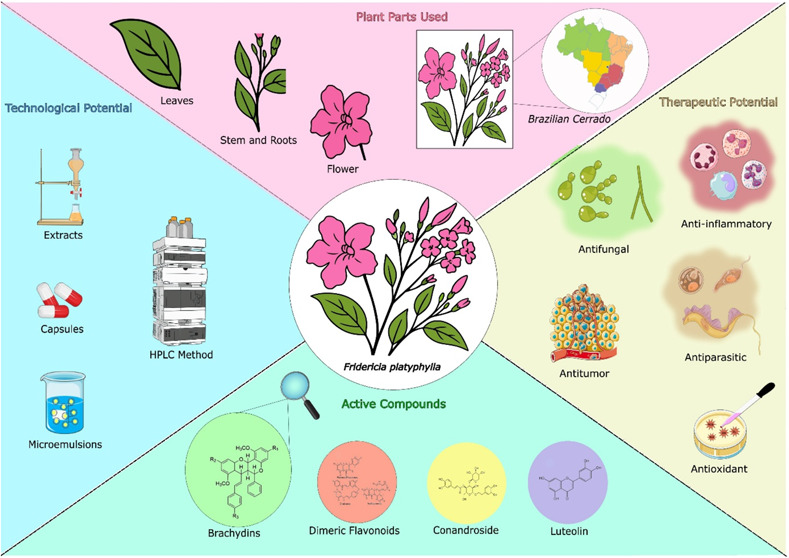

## 1 Introduction

Plant species rich in flavonoids have proven to be a potential alternative in bioprospecting for bioactive compounds that act in various pathologies, as these species possess anti-inflammatory, antioxidant, and antimicrobial properties.^1–4^ One of these plant species with potential for therapeutic use is *Fridericia platyphylla* (Cham.) L.G. Lohmann or its synonym *Arrabidaea brachypoda* (DC.) Bureau, also popularly known as “cervejinha do campo”, “cipó-una”, or “tintureiro”.^1,5,6^

The genus Fridericia Mart., Bignoniaceae, comprises climbing plants native to Tropical America, ranging from Mexico to Argentina, with several species found predominantly in the Brazilian Cerrado. These plants have been traditionally used for diverse therapeutic purposes, including astringent, antioxidant, anti-inflammatory, antimicrobial, antitumor, and wound-healing applications.^7–10^ In traditional Brazilian medicine, particularly in the Southeast and Northeast regions, the roots and leaves of *F. platyphylla* are used to treat kidney stones and joint pain.^11^ Among the species in the genus, *F. platyphylla* stands out for its chemical diversity, with at least 29 isolated substances, predominantly flavonoids, followed by terpenes, which are widely known for their pharmacological relevance.^12,13^

Flavonoids, as a significant class of phenolic secondary metabolites, are broadly distributed throughout the plant kingdom, with over 4,200 identified structures and well-established biological activities. Compared to other species, *F. platyphylla* exhibits a vibrant and diverse phytochemical profile with significant pharmacological potential.^3,14^ A subclass of unusual dimeric flavonoids called brachydins (originating from the name *A. brachypoda*) was isolated from this species for the first time.^15,16^

Recent studies have reported various biological properties for *F. platyphylla* extracts obtained from distinct plant parts. The flowers are rich in chalcones and enhance the efficacy of norfloxacin through synergistic effects.^17^ The leaves contain flavonoids with estrogenic and mutagenic activities, while the branches exhibit inhibition of lipoxygenase enzymes. The roots concentrate the most potent bioactive compounds, including flavonoids, triterpenes, saponins, tannins, and other polyphenols, which confer anti-inflammatory, antinociceptive, antiproliferative, cytotoxic, estrogenic, mutagenic, gastroprotective, antileishmanial, and trypanocidal effects.^11,13,15,18–21^

In addition to these findings, our research group demonstrated the antispasmodic effect of the hydroethanolic leaf extract in isolated rat jejunum, mediated by inhibition of Ca^2+^ influx through voltage-dependent calcium channels.^19^ These results highlight the potential of *F. platyphylla* for developing bioproducts that modulate smooth intestinal muscle contractility. More recently, efforts have focused on formulating suspensions containing crude or purified extracts to enhance oral bioavailability and mitigate toxicity.

Despite the increasing number of pharmacological studies on *F. platyphylla*, significant gaps remain, particularly regarding the lack of standardized methodologies, limited toxicological data, and heterogeneity in experimental designs, which hinder comparative analysis and the translational potential of these findings. To address these challenges, this review systematically compiles and critically analyzes preclinical evidence from *in vitro* and *in vivo* models, encompassing both crude extracts and isolated compounds. By identifying consistent pharmacological patterns, mechanistic insights, and safety concerns, this work provides a consolidated foundation to guide future investigations and support the rational development of *F. platyphylla*-based therapeutic strategies.

## 2 Results

Table 1 shows the chromatographic parameters, phytochemical compounds isolated, as well detection methods, and biological activities of the studies included in the systematic review. Tables 2 and 3 highlights the main characteristics (experimental model, *in vivo* administration, strain, *in vitro* design, groups, *in vivo* monitoring, and dose or concentration administered *in vivo*/*in vitro*) and main findings obtained from the use of the crude extract or phytochemical compounds isolated from the species *F. platyphylla*.

**Table 1:** Plant Part-Based Analysis of Phytochemical Composition, Detection Methods, and Biological Activities[Table-fn t1fn1]

plant part	extraction method	structural elucidation and analytical parameters*	bioactive compounds	concentration–effect parameters	biological activity (highlights)	references
Roots	Percolation at room temperature with ethanol: water (7:3). Extracts were fractionated with dichloromethane (CH_2_Cl_2_) and methanol: water (7:3)	The active compounds were identified as dimeric flavonoids (brachydin A, B, and C). MPLC; Injection volume not specified; Stationary phase C18 (460 × 70 mm^2^, 15–25 μm); Mobile phase: MeOH + 0.002% HCOOH in H_2_O; Flow rate: 3.5 mL/min; Gradient: 5–100% MeOH over 50 h; with structures elucidated using HPLC-PDA, UV–vis, ^1^H and ^13^C NMR, COSY, NOESY, HSQC, HMBC, HRMS, Acetyl derivatization.	Brachydin B	**Concentration**: 0.24–20 μM	Focus on the antiparasitic effect against *Trypanosoma cruzi* in both *in vivo* and *in vitro* models	Rocha et al.
		Control not specified	Brachydin C	**Analysis Software**: GraphPad Prism v5.01.		
				**Positive Control**: Benznidazole (IC_50_ = 11.3 μM)	*In vitro*: based on parasite viability against *T. cruzi*. Parasites were incubated with test compounds for 24 h at 37 °C and 5% CO_2_. Viability was assessed by direct counting in a Neubauer chamber	
				**Brachydin B**: IC_50_ = 5.3; **Brachydin C**: IC_50_= 6.6 μM	*In vivo* (parasitized mice)	
				**Replicates**: Triplicate assays.	*in vitro:* LC_50_ = 15.6 μM (brachydin B), 17.3 μM (brachydin C); *in vivo* (mice): 100 mg/kg brachydin B reduced parasitemia by 92%, no apparent toxicity	
	Percolation (300 g of roots) at room temperature with ethanol/water (7:3)	09 compounds were isolated, including two phenylethanoid glycosides and seven glycosylated dimeric flavonoids, designated as brachydins D to J	N.A.	**Concentration**: 10–300 mg/kg—gavage; main effects observed at 300 mg/kg	Focus on gastroprotective effect *in vivo* model	Rocha et al.
		MPLC and HPLC-PDA-MS was performed; Injection volume not specified; Stationary phase: C18 (460 × 49 mm, 15–25 μm); Mobile phase: MeOH in H_2_O + 0.002% HCOOH over 30 h; Flow rate: 10 mL/min; Gradient: 10–100% MeOH in H_2_O + 0.002% HCOOH over 30 h; with structures elucidated using HPLC-PDA, UV–vis, NMR (^1^H, ^13^C, COSY, NOESY, HSQC, HMBC), HRMS, and ECD spectroscopy		**Analysis Software**: GraphPad Prism v5.01.	No signs of toxicity after 7 and 14 days of treatment; no adverse effects on organ weights or body weight progression	
		Control not specified		**Replicates**: 5–6 assays		
	Percolation (300 g of roots) at room temperature. with ethanol/water (7:3)	The active compounds were identified as dimeric flavonoids (brachydin A, B and C)	N.A.	**Concentrations**: 10, 30, and 100 mg/kg (oral); Main effect at 30 mg/kg	focus on nociceptive and mechanistic pain assays	Rodrigues et al.
		HPLC–PDA was performed; Injection volume: 10 μL		**Analysis Software**: GraphPad Prism v5.00.	*In vitro*: No signs of toxicity Immediate postdose. No signs of toxicity were observed at doses up to 300 mg/kg. Rotarod test confirmed absence of sedation or motor impairment. The extract was considered behaviorally safe under the tested conditions	
		Stationary phase: XBridge C18 (250 × 4.6 mm, 5 μm); Mobile phase: MeOH + 0.002% HCOOH (A) and H_2_O + 0.002% HCOOH (B); Gradient: 5–100% A over 60 min +10 min hold; Flow rate: 1 mL/min; with structures elucidated using HPLC-PDA, UV–vis (210 and 254 nm)		**Replicates**: Not performed. Each experimental group consisted of 8–10 mice.		
		Control not specified				
	Percolation (amount not specified) at room temperature with ethanol/water (7:3). Extracts were fractionated with dichloromethane (CH_2_Cl_2_)	The active compounds were identified as dimeric flavonoids (brachydin A, B and C).	Brachydin B	**Concentrations**: Against promastigote form: 0.25–20 μM (72 h); Against amastigotes form: 0.24–20 μM (72 h)	Focus on antiparasitic effect against *Leishmania* *amazonensis* *in vitro* model	Rocha et al.
		UHPLC-HRMS was performed; Column and injection volume not detailed; purity confirmed by MS-based profiling; purity >98%; with structures elucidated using		IC_50_ for brachydin B: 2.2 ± 0.09 μM (amastigotes); **Analysis Software**: GraphPad Prism v5.01.	*In vitro:* No cytotoxic effects were observed in host macrophages at concentrations up to 50 μM, as confirmed by Alamar Blue viability assay and automated nuclear staining (Hoechst 33342). Menadione was used as a positive control.	
		UHPLC-HRMS, UV–vis (Alamar Blue at 570 and 600 nm); High-Content Imaging (Hoechst 33342 fluorescence); ^1^H, ^13^C NMR and HRMS		**Replicates**: Quadruplicate assays.		
		Control not specified				
	Percolation at room temperature with ethanol/water (7:3)	10 compounds were isolated and identified as dimeric flavonoids (brachydins A to J)	N.A.	**Concentrations**: Hydroethanolic extract. Viability assays: 5–100 mg/mL	Intracellular ROS detection using CM-H2DCFDA; concentrations: 5, 30, and 60 mg/mL; time points: 1–24 h; no significant ROS modulation	Serpeloni et al.
		LC-MS dereplication was performed; column and injection volume not specified; comparison with previously characterized hydroethanolic extract. In addition, the structures were elucidated using LC-MS dereplication through detection of specific [M – H]^−^ ions in the *m*/*z* range of 573–603, consistent with previously isolated dimeric flavonoids		EC_50_: 56.16 μg/mL (GAS), 43.68 μg/mL (ACP02), 42.57 μg/mL (HepG2)	*In vitro*: Cytotoxicity was evaluated using multiple assays: MTT assay (570 nm) and Neutral Red uptake assessed cell viability; LDH release assay (340 nm) measured membrane damage; AO/EB fluorescence microscopy (515–560 nm) and Annexin V/PI flow cytometry distinguished apoptotic and necrotic cells. The extract showed selective cytotoxicity toward tumor cells, with necrosis at 30–60 mg/mL, increased NBUDs, and downregulation of BCL-XL, BIRC5, and MET, suggesting genomic instability and impaired cell survival mechanisms	
		Control not specified		**Analysis Software**: GraphPad Prism v5.01.		
				**Replicates**: Triplicate assays.		
	Percolation at room temperature with 70% EtOH. Further liquid–liquid partitioning was done using CH_2_Cl_2_ and MeOH-H_2_O (7:3)	The active compounds were identified as brachydin A, B and C and halogenated brachydins	Halogenated brachydins	**Concentrations**: Compounds were tested at concentrations of 1.25, 2.5, 5, and 10 μM for IC_50_ determination, and 100 μM for cytotoxicity screening. IC_50_ values against L. amazonensis amastigotes were 1.0 ± 0.3 μM (4,9,11-Tribromobrachydin C) and 1.2 ± 0.4 μM (11-Chlorobrachydin C); against *T. cruzi* amastigotes, 1.4 μM for both compounds 4,11-Dibromobrachydin C, 4-Iodobrachydin B, 11-Chorobrachydin B, and 11-Chlorobrachydin C. CC_50_ for macrophages was >100 μM in all cases. Selectivity indices ranged from >71 to >100. **Positive control**: Amphotericin B and benznidazole.	Focus on antiparasitic potential	Neuenschwander et al.
		UHPLC-PDA-ELSD-MS and HPLC-PDA. Full scan: 150–1000 *m*/*z*. Injection volume: 4 μm (UHPLC), 20 μL (HPLC). Stationary phase: BEH C18 (50 × 2.1 mm^2^, 1.7 μm) for UHPLC, PF C18 HQ (250 × 4.6 mm, 10 μm) for HPLC. Mobile phase: MeCN/H_2_O both with 0.1% formic acid. Gradient (UHPLC): 5–98% MeCN in 4.0 min, held 0.8 min, re-equilibrated; flow rate: 600 μL/min (UHPLC), 1 mL/min (HPLC). UV detection at 254 and 280 nm; with structures elucidated using UHPLC-PDA-ELSD-MS, HRESIMS, and 1D/2D NMR (^1^H, ^13^C, HMBC, ROESY). Halogenation patterns were confirmed by aromatic shift analysis and HMBC/ROESY correlations		**Analysis software**: Not specified.	*In vitro*: Based on viability of mouse peritoneal macrophages exposed to halogenated biflavonoids derived from A. brachypoda. Cytotoxicity was assessed by resazurin metabolism assay (AlamarBlue). None of the tested compounds (4–19) showed toxicity at the maximum concentration of 100 μM; CC_50_ values were all >100 μM	
		Control not specified		**Replicates**: Assays were performed in triplicate (three biological replicates).		
	Hydroethanolic extraction (70% ethanol), followed by fractionation with dichloromethane. Compounds were diluted in DMSO and PBS to a final concentration of 0.5% DMSO for cell culture assays	The active compounds were identified as brachydin A, B and C	Brachydin A	**Concentration**: Cells were treated with nine concentrations of each compound (0.24 to 30.72 μM) for 24 h. IC_50_ values in PC-3 cells were 23.41 μM (brachydin A), 4.28 μM (brachydin B), and 4.44 μM (brachydin C).	Focus on the antioxidant assay and antitumor activity. *In vitro*: All compounds showed cytotoxicity against PC-3 prostate cancer cells. Brachydin B and brachydin C, (0.96–6 μM) for 1–24 h, induced both apoptosis and necrosis, while brachydin A (6 μM, 24 h) predominantly induced necrosis at higher concentrations. Elevated cleaved PARP expression supported apoptosis. Brachydin B and brachydin C upregulated p21, suggesting G1 arrest; brachydin A and brachydin B decreased phospho-AKT levels. No genotoxicity was observed in the comet assay	Nunes et al.
		UHPLC-HRMS was performed. Structural elucidation was performed using NMR and HRMS	Brachydin B	**Analysis Software**: Statistical analysis was performed using RStudio v1.1.442. ImageJ was used for densitometric analysis of Western blots, and Comet Imager v2.2 was used for comet assay image analysis.		
		Control not specified	Brachydin C	**Replicates**: Three biological and four technical replicates were used for viability assays.		
	Two extracts were prepared: a hydroethanolic extract of *A. brachypoda* (HEAB) and a dichloromethane fraction of *A*. *brachypoda* (DCMAB)	The active compounds were identified as brachydin A, B and C	Brachydin A	**Concentration**: HEAB and DCMAB were tested at 3–100 μg/mL; brachydins A, B and C at 3–100 μM. IC_50_ values for IL-6 inhibition were: 62 ± 2 μM (brachydin A), 17 ± 3 μM (brachydin B), 19 ± 3 μM (brachydin C); and 31 μg/mL for DCMAB. HEAB showed no significant effect (IC_50_ > 100 μg/mL)	Focus on anti-inflammatory effects in an osteoarthritic inflammation model	Salgado et al.
		HPLC-PDA and UHPLC-MS/MS were employed for analysis and quantification. The HPLC-PDA method used a Waters X-Bridge C18 column (250 × 4.6 mm^2^, 5 μm), gradient elution (5–100% MeOH with 0.1% FA), flow rate 1 mL/min, and detection at 254 nm. Quantification was performed by UHPLC-MS/MS (BEH C18 column, 50 × 2.1 mm^2^, 1.7 μm) using MRM mode. Calibration curves ranged from 31 to 500 ng/mL with *r* ^2^ > 0.99	Brachydin B	**Analysis Software**: GraphPad Prism 8.3.	*In vitro*: Cytotoxicity was assessed using the WST-1 assay. DCMAB showed concentration-dependent cytotoxicity to HFLS. HEAB was noncytotoxic up to 100 μg/mL. Among the isolated compounds, brachydin B and C reduced HFLS viability at 50 μM, while 1 was cytotoxic only at 100 μM	
		Control not specified	Brachydin C	**Replicates**: Experiments were run in six replicates (two experiments per donor, three donors; *n* = 6)		
	Percolation with 70% ethanol. The extract was evaporated under reduced pressure at a temperature below 40 °C and then lyophilized. The crude extract underwent liquid–liquid partitioning with CH_2_Cl_2_ and H_2_O/MeOH (7:3). The dichloromethane phase was fractionated on a silica gel column using hexane/ethyl acetate and ethyl acetate/methanol gradients, yielding 19 fractions. Compounds were further purified based on HPLC-PDA and TLC profiles.	Brachydins A, B, and C were identified by comparison with previously isolated and characterized standards. HPLC-PDA and TLC. Structural identity was established in earlier studies *via* NMR and HRMS. Column chromatography: silica gel 60 (0.063–0.200 mm, Merck) as the stationary phase. A linear polarity gradient of hexane/ethyl acetate to ethyl acetate/methanol was applied.	Brachydin A	**Compounds**: Electrochemical analyses were performed using brachydins A, B, and C at a fixed concentration of 0.300 mmol L^–1^. Solutions were prepared in methanol and diluted with 0.04 mol L^–1^ Britton-Robinson buffer containing 0.1 mol L^–1^ KCl and 20% methanol to ensure solubility	Focus on antioxidant assay using electrochemical behavior. Electrochemical oxidation profiles were used as an indirect measure of antioxidant potential. Differential pulse voltammetry revealed that brachydin A had the lowest oxidation potential (+0.48 V), followed by brachydin C (+0.57 V) and brachydin B (+0.71 V). Oxidation peak potentials shifted to less positive values with increasing pH	Nascimento et al.
			Brachydin B	**Analysis Software**: GPES software (Eco Chemie, Autolab PGSTAT 302N system)		
			Brachydin C	**Replicates**: The number of replicates was not explicitly stated		
	Hydroethanolic extraction, followed by liquid–liquid partitioning with dichloromethane (CH_2_Cl_2_) and H_2_O-MeOH (7:3)	The isolated compounds were Brachydin E and Brachydin F	Brachydin E	**Concentrations**: 1.6, 3.12, 6.25, 12.5, 25, 50, and 100 μg/mL. IC_50_ values for PC-3 cells were 5.9 ± 1.3 μg/mL for Brachydin E and 33.1 ± 7.4 μg/mL for Brachydin F.	Focus on antiproliferative, cytotoxic, and pro-apoptotic activities	Lima et al.
		HPLC-PDA, NMR and HR-ESI-MS. HPLC was conducted using a reverse-phase C18 column with gradient elution of water (0.1% formic acid) and acetonitrile (0.1% formic acid), at a flow rate of 0.5 mL/min. Detection was achieved *via* PDA at 254 nm. MS analysis was performed using electrospray ionization (ESI) in positive ion mode. ESI-MS revealed [M + Na]^+^ ions at *m*/*z* 883 and 913, and high-resolution electrospray ionization mass spectrometry (HR-ESIMS) confirmed [M – H]^−^ ions at *m*/*z* 859.2457 and 889.2586.	Brachydin F	**Positive control**: Doxorubicin at concentrations of 0.16, 0.31, 0.62, 1.25, 2.5, 5, and 10 μg/mL (100 μL/well)	*In vitro:* Brachydins E and F showed selective cytotoxicity against PC-3 (prostate) tumor cells without affecting HaCaT (nontumoral) cells at similar concentrations, suggesting low systemic toxicity	
		Control not specified		**Analysis Software**: IC_50_ values were calculated *via* linear regression using Origin software.		
				**Replicates**: Assays were performed in triplicate, in three independent experiments.		
	Percolation at room temperature with ethanol/water (7:3). Extracts were fractionated with dichloromethane (CH_2_Cl_2_) and methanol:water (7:3). The purified compound (≥98%) was lyophilized. The stock solution was prepared in DMSO at –20 °C	Brachydin A was the only compound investigated	Brachydin A	**Concentrations**: Brachydin A was tested at concentrations ranging from 10 to 100 μM. Cytotoxic effects were observed from 60 μM at 48 h, and 40 μM at 168 h. IC_50_ values were not explicitly calculated in this study	Focus on Antioxidant assay. Antioxidant activity was indirectly assessed *via* high-content screening using the mitochondrial superoxide indicator MitoSOX Red, which measures ROS production. Brachydin A did not significantly increase MitoSOX Red fluorescence, indicating it did not promote mitochondrial ROS generation under the tested conditions.	Ribeiro et al.
		The article does not describe the chromatographic method used for BrA isolation in this study, nor does it mention the use of commercial reference standards. All compound-related details, including purity, were obtained from previous work		**Positive control**: Docetaxel	*In vitro*: Toxicity was evaluated using DU145 metastatic prostate tumor spheroids. Brachydin A decreased cell viability in a time- and dose-dependent manner, starting from 40 μM at 48 h. Flow cytometry showed increased apoptosis and necrosis, with >61% apoptosis at concentrations ≥80 μM. High-content screening revealed mitochondrial depolarization. Western blotting confirmed increased markers of DNA damage (cleaved-PARP, p-γ-H2AX), apoptosis (CASP3, CASP7, CASP8), and inflammation (NF-kB, TNF-α), suggesting brachydin A induces cell death by PARP-related	
				**Negative control**: RPMI 1640.		
				**Solvent control**: DMSO 1%.		
				**Analysis Software**: GraphPad Prism 7.0 software.		
				**Replicates**: All assays were conducted with six replicates (*n* = 6) per group in three independent experiments (*n* = 3)		
	Percolation at room temperature with ethanol/water (7:3). Extracts were fractionated with dichloromethane (CH_2_Cl_2_) and methanol:water (7:3).The extraction process involved lyophilization, and the compound was dissolved in dimethyl sulfoxide (DMSO) and PBS for experimental use	No chromatographic method or analytical validation using reference standards was detailed in this study. The compound brachydin B was characterized and provided by collaborating institutions based on prior studies	Brachydin B	**Concentrations**: Brachydin B showed cytotoxicity at concentrations ≥ 1.50 μM after 24 h (MTT assay), with an IC_50_ value of 7.45 μM in 2D models. In 3D spheroids, cytotoxicity was observed at concentrations ≥ 50 μM after 48 h, and ≥30 μM after 168 h.	Focus on antitumoral, antiproliferative, and antimigratory effects of brachydin B	Sperloni et al.
				**Positive control**: Docetaxel	*In vitro*: Brachydin B demonstrated selective cytotoxicity toward DU145 cancer cells without affecting nontumoral HGF cells at low doses. In 2D culture, cytotoxicity was confirmed *via* MTT, LDH release, and triple staining, which showed no signs of apoptosis/necrosis at lower concentrations. In 3D spheroids, brachydin B inhibited viability and volume growth at high concentrations and long exposure times. No *in vivo* toxicity assessment was performed	
				**Negative control**: PBS.		
				**Analysis Software**: GraphPad Prism 7.0. TScratch software; AxioVision SE64 Rel. 4.9.1.		
				**Replicates**: All experiments were performed in biological triplicates (*n* = 3)		
	Percolation at room temperature with 70% ethanol. The resulting hydroethanolic extract was evaporated, lyophilized, and fractionated *via* liquid–liquid partition using dichloromethane and methanol–water (7:3). The dichloromethane fraction (FDCM) was selected for microemulsion incorporation	Three dimeric flavonoids—brachydins A, B, and C—were identified. The FDCM was analyzed using HPLC-UV/PDA with a C18 column (5 μm, 150 × 4.6 mm^2^, 100 Å). A gradient mobile phase of methanol and water (both acidified with 0.01% formic acid) was used at a flow rate of 1 mL/min, with UV detection at 254 nm. No external reference standards were specified; identification was based on UV spectra and retention times	N.A.	**Concentrations**: FDCM was incorporated into microemulsions (ME) at 3% (ME3) and 5% (ME5) w/w. Particle size (DLS: 36.7–75.4 nm), PDI (0.248–0.604), and ζ-potential were measured. Release kinetics used Franz cells (6 h; 63.5% cumulative release for ME3). The study did not report IC_50_ or IG_50_ values	Focus on physicochemical stability, *in vitro* release, and *in vivo* toxicity	Nascimento et al.
		Control not specified		**Negative control (** ***in vivo*** **)**: trauma and DMSO	*In vivo*: toxicity was evaluated using *Tenebrio molitor* larvae injected with ME3 or FDCM (1–100 μg/mL). No toxicity or behavioral changes were observed over 7 days	
				**Software Analysis**: GraphPad Prism 6 and DDsolver.	Survival was 100% in all treated groups, indicating the safety of both the microemulsion and the isolated fraction	
				**Replicates**: 3 (triplicate) for physicochemical assays, *in vitro* release, and stability studies.		
	Maceration at room temperature with ethanol/water (7:3). Extracts were fractionated with dichloromethane (CH_2_Cl_2_) and methanol:water (7:3). The crude DCM and aqueous-methanolic fractions were obtained after decantation and evaporated under a vacuum at approximately 40 °C	The DCM fraction enriched with brachydin A was analyzed using HPLC-UV/PDA-MS with a C18 column (5 μm, 150 × 4.6 mm^2^, 100 Å). A gradient mobile phase of methanol and water, both acidified with 0.01% formic acid, was employed at a flow rate of 1 mL/min, with UV detection at 254 nm. No external reference standards were specified; identification was based on UV spectra and retention times.	Brachydin A	**Concentrations**: The encapsulation of the brachydin A compound into micelles formed by the F127 copolymer was carried out using the solid dispersion method. This process resulted in the formation of brachydin-loaded micelles at final concentrations of 0.5%, 0.25%, or 0.125% (w/v) of F127, with a fixed brachydin A concentration of 500 μg/mL	Focus on physicochemical characterization, encapsulation efficiency, *in vitro* release kinetics, leishmanicidal activity, and cytotoxicity	Costa et al.
		Control not specified		**Control**: Micelles containing only F127 were prepared in parallel and used as controls in all experiments	*In vitro*: The study evaluated the physicochemical properties (particle size, Polydispersity Index, ζ-potential), drug release kinetics using Franz diffusion cells, and antileishmanial activity against *L. amazonensis* promastigotes. In addition, brachydin A demonstrated selective cytotoxicity in mouse macrophage cell line (RAW 264.7)	
				**Replicates**: All the measurements were performed in triplicate		
				**Software Analysis**: ORIGIN 10.0		
	Percolation at room temperature with ethanol:water (7:3). The crude extract was subjected to liquid–liquid partitioning with CH_2_Cl_2_ and H_2_O/MeOH (7:3) to obtain DCMF	The DCM fraction enriched with brachydins A, B, and C was analyzed using HPLC-PDA and LC-MS. Nanoparticles were characterized by DLS, ζ-potential, AFM, FTIR, and DSC. Chromatographic conditions were not described in detail in this study, but are available in a previous work (Da Rocha et al., 2017). No external reference standards were specified; identification was based on retention profiles and published data on brachydin content	Brachydin A, Brachydin B, Brachydin C	**Concentration**: Promastigote and amastigote assays: 0.06 to 125 μg/mL	Focus on physicochemical characterization, encapsulation efficiency, stability, leishmanicidal activity, and cytotoxicity	Neves et al.
				**Cytotoxicity assay**: up to 500 μg/mL	*In vitro:* ZNP-DCMF exhibited potent activity against *L. amazonensis*, with IC_50_ = 36.33 μg/mL (promastigotes) and 0.72 μg/mL (amastigots). In contrast, nonencapsulated DCMF showed IC_50_ = 253.1 μg/mL and 6.96 μg/mL, respectively. The formulation demonstrated high selectivity (SI = 694.44) and low cytotoxicity in RAW 264.7 macrophages (CC_50_ > 500 μg/mL). Physicochemical analysis confirmed a mean particle size of 206 nm, PDI < 0.2, encapsulation efficiency >99%, and 49-day stability	
				**–Negative controls**: 1% DMSO; blank zein nanoparticles		
				**Replicates**: All experiments were performed in triplicate		
				**Analysis Software**: GraphPad Prism 9.0		
Leaves Stalks and Roots	Percolation at room temperature with ethanol/water (7:3)	The active compounds were identified as dimeric flavonoids (brachydin A, B and C).	Brachydin A	**Concentration**: RYA assay—Increasing concentrations from 10 to 300 μg/mL.	Focus on estrogenic and mutagenic assays	Resende et al.
		HPLC was performed; Injection volume not specified; Stationary phase: C18 (150 × 49 mm^2^, 5 μm); Mobile phase: A gradient of methanol (MeOH) and water (H_2_O), both containing 0.002% formic acid (HCOOH), from 5 to 100% MeOH over 60 min. Flow rate: 10 mL/min; with structures elucidated using HPLC-PDA, UV–vis, ^1^H and ^13^C NMR, and Direct MS	Brachydin B	**Ames test**: 1, 10, 50, 100, and 500 μg/plate	*In vitro*: Estrogenicity (RYA): EC_50_ = 56.2 μg/mL (leaves), 191.3 μg/mL (roots); Estradiol Equivalent Concentration (EEQ): EEQ 7.4 ± 2.3 nM (leaves), EEQ 2.16 ± 0.9 nM (roots). Estrogenic activity mediated by ERα	
		Control not specified	Brachydin C	**Controls used**: **Positive controls**: 4-nitro-*o*-phenylenediamine (NPD), sodium azide, mitomycin C, 2-anthramine	Mutagenic activity in TA98 (Ames test); DNA damage potential; flavonoids implicated	
				**Negative control**: DMSO		
				With and without metabolic activation (±S9 mix)		
				**Analysis Software**: GraphPad Prism v5.00		
				**Replicates**: 6 experiments in triplicate		
Aerial parts - branches	Maceration with ethanol (P.A) at room temperature. Solvent removed under reduced pressure. solid-phase extraction with CH_3_OH-H_2_O (30, 50, 100%)	Conandroside (phenylethanoid glycoside)	Conandroside	**Concentrations**: Crude ethanol extract: 25 μg/mL	Focus on anti-inflammatory activity	Bertanha et al.
		HPLC-DAD and HPLC-HRMS were performed. Injection volume not specified. Stationary phase: C18 column (Onyx Monolithic 100 × 10 mm^2^); mobile phase: CH_3_OH-H_2_O + 0.1% acetic acid; gradient 5–100% MeOH in 30 min; flow rate 4 mL/min; HRMS: ESI-TOF mode with methanol gradient 95% H_2_O to 100% CH3OH over 35 min. In addition, the structure was detected and characterized by HPLC-DAD, HRMS, ^1^H and ^13^C NMR		Conandroside and quercetin were evaluated at concentrations ranging from 1.25 to 80 μM	*In vitro* (normal human fibroblasts, GM07492A): CC_50_ > 2500 μM (conandroside); extract: CC_50_ = 2352.0 ± 28.5 μg/mL. The data shows that both crude ethanol extract and conandroside are not presented as cytotoxic	
		Control not specified		Crude ethanol extract-IC_50_: 49.4 ± 2.5 μg/mL		
				Conandroside- IC_50_: 7.8 ± 1.1 μM; Quercetin (control) -IC_50_: 7.6 ± 0.3 μM		
				**Analysis Software**: not specified		
				**Replicates**: triplicate assays		
Leaves	Percolation at room temperature with ethanol/water (7:3). Crude extract evaporated under vacuum (∼40 °C); liquid–liquid partitioning with hexane, ethyl acetate, and methanol/water (7:3)	HPLC-UV/vis and MS/NMR.Injection volume not specified. Stationary phase: Silica gel 60 (0.063–0.200 mm)	Luteolin	**Concentrations**: Luteolin: 5, 9.7, 19.5, 39.2, 78.5, 157–314 μM	Focus on antiproliferative and pro-apoptotic activity	Franco et al.
		Mobile phase: Gradient elution with hexane:ethyl acetate followed by ethyl acetate:methanol. Gradient: Increasing polarity with two-step solvent system (hexane/EtOAc, then EtOAc/MeOH); no proportions or timing provided. Control not specified		**U-251 (glioblastoma)**- IG_50_: 6.6 ± 1.3 μM	*In vitro*: U-251 cells were the most sensitive to luteolin, followed closely by HaCaT and NCI-H460, while HT-29 and 786–0 cells showed the highest resistance. Selectivity Index (SI) ≈ 0.98 (U-251 *vs* NHA), suggesting low selectivity between tumor and nontumor cells	
				**NCI-ADR/RES (resistant ovarian cancer)-** IG_50_: 15.0 ± 1.3 μM		
				**NCI-H460 (lung cancer)**-IG_50_: 8.0 ± 0.3 μM		
				**786–0 (kidney cancer)**-IG_50_: 55.2 ± 9.7 μM		
				**PC-3 (prostate cancer)**-IG_50_: 26.2 ± 2.0		
				**HT-29 (colon cancer)**- IG_50_: 60.8 ± 11.1 μM		
				**HaCaT (nontumoral** keratinocyte line)-IG_50_: 7.6 ± 2.4 μM		
				**Analysis Software**: ORIGIN 8.0 and GraphPad Prism		
				**Replicates**: Triplicate assays		
Flowers	Percolation at room temperature with ethanol/water (7:3). Extracts were fractionated with dichloromethane (CH_2_Cl_2_)	The active compounds were identified as dimeric flavonoids (brachydin A, B and C)	Brachydin A	**Concentrations**: MIC assay: 8–512 μg/mL; Norfloxacin/EtBr modulation at 128 and 256 μg/mL (1/8 and 1/4 MIC)	Focus on antimicrobial activity and Norfloxacin modulation (inhibition of the Norfloxacin efflux pump).	Andrade et al.
		HPLC-PDA was performed; Injection volume: 10 μL. Stationary phase: Luna C18 (250 × 4.6 mm^2^, 5 μm); Mobile phase: A (2% acetic acid in H_2_O), B (2% acetic acid in MeOH); Gradient: 5–95% B over 30 min; Flow rate: 1 mL/min; In addition, the structures were elucidated using LC-ESI-MS analysis provided *m*/*z* fragments ranging from 573 to 603 [M – H]^−^. HPLC-PDA, UV–vis (210–254 nm)	Brachydin B	**Analysis Software**: GraphPad Prism v5.00	*In vitro*: antimicrobial activity of floral extracts rich in brachydins A, B, and C was evalueted. The Minimum Inhibitory Concentration (MIC) was determined over a concentration range of 8–512 μg/mL. Additionally, efflux pump modulation was assessed using norfloxacin combined with ethidium bromide at subinhibitory concentrations (128 and 256 μg/mL), indicating interference with bacterial resistance mechanisms	
		Control not specified	Brachydin C	**Replicates**: triplicate assays		
	Hydrodistillation using a Clevenger apparatus for 3 h; organic phase washed with dichloromethane and dried with anhydrous sodium sulfate	15 compounds identified; major: trans-anethole (11.10%), β-thujene (14.87%), 3-carene (21.07%), γ-terpinene (32.01%)	N.A	**Concentrations**: 1.0, 2.0, 4.0, 6.0, 8.0, 10.0, 20.0, 30.0, 40.0 e 50.0 μL/mL. IC_50_ value not calculated; **Analysis Software**: PAST 3.0	*Focus on antioxidant potential*	Menezes-Filho
		GC-MS was performed. Injection volume not specified. Stationary phase: Restek Rtx-5 ms capillary column (30 m × 0.25 mm × 0.25 μm), nonpolar		**Replicates**: triplicate assays	*In vitro:* The essential oil extracted presented 15 volatile compounds—mainly γ-terpinene and 3-carene, both known for their antioxidant properties. Antioxidant activity was assessed using the DPPH radical scavenging assay. The extract showed dose-dependent radical scavenging activity across concentrations from 1.0 to 50.0 μL/mL, indicating relevant antioxidant potential likely attributed to its high content of monoterpenes	
		Mobile phase: Helium (carrier gas) at 57.4 kPa. Temperature gradient: 60 °C (3 min), then +3 °C/min to 200 °C, then +15 °C/min to 280 °C (1 min hold). Injector/Detector: temperatures: 230/300 °C. Ionization mode: Electron Impact (EI) at 70 eV. Detection by GC-MS (mass range: 43–550 *m*/*z*). and comparison with Kovats Index and NIST 11 library.				
		DPPH radical scavenging assay in 96-well microplate; absorbance read at 517 nm; incubation at 1 °C for 1 h in the dark.				
		Control not specified				
	The flowers were macerated in 1 L of 70% hydroethanolic solution for 48 h at room temperature in amber glass containers. After filtration, the extract was concentrated under reduced pressure, frozen at –12 °C, and lyophilized. The lyophilized extract was stored at –12 °C until further use	No specific compounds were isolated or identified	N.A.	**Concentration**: The extract was tested at concentrations of 500, 250, 125, 62.5, 31.25, and 15.62 mg/mL. Inhibition halos ranged from 26.1–9.1 mm for *Candida albicans*, 19.7–4.6 mm for *Candida guilliermondii*, 16.9–6.4 mm for *Candida tropicalis*, and 11.8–8.7 mm for *Candida krusei*. **Positive control**: Cetoconazole (50 μg/mL) **Negative control**: DMSO	Focus on antifungal evaluation	Menezes-Filho, Porfiro e Castro
		No chromatographic profiling or reference standard quantification was performed. Only organoleptic (color, aroma, clarity), pH (5.25 ± 0.04), and relative density (0.9699 g/mL at 20 °C) parameters were reported for extract characterization		**Analysis Software**: Statistical analysis was conducted using the Scott-Knott test at a 5% significance level. No software was specified in this study	*In vitro:* The extract showed antifungal activity against *Candida* species, with inhibition halos ranging from 26.1 mm (*C. albicans*) to 4.6 mm (*C. guilliermondii*), depending on the concentration (15.62 to 500 mg/mL). *C. albicans* was the most sensitive, while *C. krusei* and *C. tropicalis* showed moderate inhibition. The antifungical activity was dose-dependent	
				**Replicates**: All assays were performed in triplicate.		
	Flowers were macerated in 1 L of 70% hydroethanolic solution for 72 h at room temperature. Extracts were concentrated under reduced pressure using a rotary evaporator and dried in a forced-air oven at 35 °C	No specific compounds were isolated or identified. No chromatographic or phytochemical profiling was performed. No internal or external standards were used for compound identification or quantification. Only the crude extracts were evaluated for biological activity	N.A.	**Concentrations**: 100, 50, 25, and 12.5 mg/mL for antifungal assays. The largest inhibition zones were 18.6 mm (F. platyphylla, C. albicans) and 14.3 mm (Fridericia florida, C. krusei) at 100 mg/mL. Cytotoxicity (LC_50_) against *Artemia salina* was 237.81 μg/mL for *F. platyphylla* and 301.20 μg/mL for *F. florida*	Focus on antifungal and cytotoxic evaluations	Menezes-Filho
				**Positive control**: Ketoconazole (50 μg/mL; antifungal assay). Potassium dichromate (cytotoxicity assay)	*In vitro*: Cytotoxicity was assessed through the brine shrimp lethality test (*A. salina*). Both floral extracts showed growth inhibiting activity for most strains, indicating biologically relevant cytotoxic potential. No *in vivo* toxicological assays were conducted	
				**Negative control**: 70% hydroethanolic solution (antifungal assay). 5 mL seawater +100 μL DMSO (cytotoxicity assay)		
				**Analysis Software**: LC_50_ was calculated *via* best-fit line using Assistat Software Free. Statistical analysis was performed using ANOVA and the Scott-Knott test at 5% significance.		
				**Replicates**: Assays were conducted in quadruplicate		

a Prepared by the authors from the PubMed, Scielo, and Google Scholar databases. **Legend**: ACP02—Human gastric adenocarcinoma cell line; AFM—Atomic Force Microscopy; CC_50_—Cytotoxic Concentration 50%; CH_2_Cl_2_—Dichloromethane; COSY—Correlation Spectroscopy; DAD—Diode Array Detector; DLS—Dynamic Light Scattering; DMSO—Dimethyl Sulfoxide; DNA—Deoxyribonucleic Acid; DPV—Differential Pulse Voltammetry; DSC—Differential Scanning Calorimetry; DU145—Human metastatic prostate cancer cell line; ECD—Electronic Circular Dichroism; EC_50_—Effective Concentration 50%; EEQ—Estradiol Equivalent Concentration; ELSD—Evaporative Light Scattering Detector; EtOH—Ethanol; EtOAc—Ethyl Acetate; FA—Formic Acid; FGH—Human Gingival Fibroblasts; GC-MS—Gas Chromatography–Mass Spectrometry; GAS—Primary human gastric cells; H_2_O—Water; HaCaT—Human keratinocyte cell line; HCOOH—Formic Acid; HMBC—Heteronuclear Multiple Bond Correlation; HPLC—High Performance Liquid Chromatography; HRMS—High Resolution Mass Spectrometry; HSQC—Heteronuclear Single Quantum Coherence; IC_50_—Inhibitory Concentration 50%; IG_50_—Inhibitory Growth 50%; LDH—Lactate Dehydrogenase; LC-MS—Liquid Chromatography–Mass Spectrometry; ME3—Microemulsion 3%; MeOH—Methanol; MET—Mesenchymal Epithelial Transition factor gene; MIC—Minimum Inhibitory Concentration; MTT—3-(4,5-dimethylthiazol-2-yl)-2,5-diphenyltetrazolium bromide; NBUDs—Nucleoplasmic Bridges and Nuclear Buds; NHA—Normal Human Astrocytes; NOESY—Nuclear Overhauser Effect Spectroscopy; NMR—Nuclear Magnetic Resonance; PARP—Poly (ADP-ribose) polymerase; PBS—Phosphate Buffered Saline; PDA—Photodiode Array Detector; PDI—Polydispersity Index; PC-3—Human prostate cancer cell line; RMS—Root Mean Square; ROS—Reactive Oxygen Species; RP-HPLC—Reverse Phase High Performance Liquid Chromatography; RPMI 1640—Roswell Park Memorial Institute medium; RYA—Recombinant Yeast Assay; SI—Selectivity Index; TA98—*Salmonella typhimurium* strain for Ames test; TLC—Thin Layer Chromatography; TScratch—Software for scratch wound healing assay analysis; UHPLC—Ultra High Performance Liquid Chromatography; UV–vis—Ultraviolet–Visible Spectroscopy; WST-1 —Water-soluble tetrazolium salt 1.

**Table 2:** Experimental Design and Main Outcomes of *In Vitro* Studies with *F. platyphylla* ^a^

experimental model	study design	groups	concentration	main findings	references
Cytotoxicity in macrophages	Culture of macrophage cells obtained from BALB/c mice	Control, Benznidazole, Amphotericin B, brachydin A, brachydin B, and brachydin C (*n* = 6)	100 mg/kg (*in vivo*); 1.23–100 μg/mL (*in vitro*)	Brachydin B and C reduced parasitemia in *T. cruzi*	Rocha et al.
*M*utagenicity and estrogenic activity	Mutagenic activity: *S. typhimurium*. Estrogenic activity: *Saccharomyces cerevisiae* strains	G1: *A*. *brachypoda* leaf extract.	3 a 24 mg per culture plate	Extracts showed mutagenic activity; leaf and root extracts had significant estrogenic activity	Resende et al.
		G2: *A*. *brachypoda* stem extract.			
		G3: *A*. *brachypoda* root extract.			
Effect against *L. amazonensis*	Macrophages from BALB/c	Control, Amphotericin B and brachydin B	*In vitro:* dose de 0.25 a *20 μM*	Brachydin B was effective against *L. amazonensis* promastigotes with no significant toxicity	Rocha et al.
Antimicrobial and drug-resistance modulation	*Staphylococcus aureus*; *Escherichia coli* and *C. albicans*	Control group: Chlorpromazine (CPZ); Crude extract of *A. brachypoda* leaves (FLAB-Et) extract; Dichloromethane fraction of *A. brachypoda* leaves (FLAB-DCM) extract; Brachydin A; Brachydin B and associations with Norfloxacin and Ethidium bromide	They were prepared in DMSO, followed by dilution in sterile water to a fin-0-al concentration of 1024 μg/mL	FLAB-Et, FLAB-DCM, and brachydin B enhanced antibiotic efficacy against resistant bacteria	Andrade et al.
Cell viability assay using MTT test was used	Tumor cells (ACP02) and normal human gastric primary cells obtained by biopsy (GAS) were used	Cytotoxic activity: *F. platyphylla* extracts in different concentrations	*F. platyphylla* root extracts (5–500 mg/mL) for 24 h	*F. platyphylla* root extracts have cytotoxic and antiproliferative effects by inducing necrosis and reducing expression of apoptosis (BCL-XL, BIRC5) and cell cycle (MET) genes	Serpeloni et al.
		Apoptotic activity: *F. platyphylla* roots extracts (5.0, 30, or 60 mg/mL) or vehicle (PBS) and doxorubicin (DXR 0.2 mg/mL).			
15-LOX inhibition assay and cytotoxicity test	Enzymatic assay with human cells in culture	Control group (enzymatic assay): Quercetin; Control group (cytotoxicity): Doxorubicin	Use of 25 μg/mL for enzymatic and cytotoxicity assays	*F. platyphylla* extract inhibited 15-LOX without cytotoxicity, and conandroside showed strong LOX inhibition potential	Bertanha et al.
Antiproliferative activity of luteolina	Culture of tumor cells of U-251 glioblastomas and cultures of nontumor cells HaCaT and NHA.	Control group: Dimethyl sulfoxide (DMSO) and Temozolomide (TMZ). Luteolin compound group	For antitumor activity, concentrations of 314, 157, 78.5 39.2, 19.5, 9.7 e	Luteolin reduced proliferation in U-251 glioblastoma cells with low toxicity to nontumor cells	Franco et al.
			5 μM were used		
Phytopathological antifungal activity	Phytopathological antifungal activity—*Sclerotinia sclerotiorum*, Colletotrichum acutatum and *Aspergillus flavus* strains; Antifungal activity in *Candida* strains - *C.* *albicans*; *C. guilliermondi*, *C. krusei* and *C. tropicalis*	Phytopathological antifungal activity: Negative control - untreated and DMSO; Positive control - Frowncide 500 SC; Essential oil (EO) tested at various concentrations	For phytopathological antifungal activity, doses of 100 (EO); 50; 25; 12.5; 6.25; 3.13; and 1.56 μL/mL of EO were used; Antifungal activity for Candida strains at concentrations (2, 4, 6 and 8% of EO) and antioxidant activity by DPPH at concentrations 50; 40; 30; 20; 10; 8.0; 6.0; 4.0; 2.0 and 1.0 μm/mL	EO inhibited fungal growth with high antioxidant activity at higher concentrations	Menezes-Filho
		Antifungal activity for *Candida*: Negative control—Tween 80; Positive control- Ketoconazole *(50 μg/mL)*; Essential oil tested at various concentrations			
Halogenated flavonoids for antiparasitic effects	Mouse macrophages infected with *L. amazonensis* and *T. cruzi*	Antiparasitic: halogenated brachydins, at different concentrations	Oxidative halogenation was performed with 50 mg of the dichloromethane fraction (DCM), obtaining 16 derivatives	Halogenated brachydins showed high selectivity for *L. amazonensis* especially compounds 4,9,11-Tribromobrachydin C and 11-Chlorobrachydin C. In addition, was observed best activity for *T. cruzi* in 4,11-Dibromobrachydin C, 4-Iodobrachydin B, 11-Chorobrachydin B, and 11-Chlorobrachydin C	Neuenschwander et al.
		Group 1: amastigote forms of *L. amazonensis*, using halogenated brachydins	Antiparasitic activity and IC_50_: 10, 5, 2.5, and 1.25 μM		
		Group 2: amastigote forms of *T. cruzi*, using halogenated brachydins			
Cytotoxicity on prostate cancer	Human prostate cancer cell line PC-3 to evaluate the cytotoxic effects of brachydins	Control (PBS, Doxorubicin), brachydins A, B, C	Concentrations of 0.24 to 30.72 μM of brachydin A, B and C were used to determine cell viability and cytotoxic effects on PC-3 prostate cancer cells. Then, doses of 1.5, 3.84, and 6 μM were administered	Brachydins showed cytotoxicity in PC-3 cells, affected cell cycle genes, and modulated the PI3K/AKT/mTOR pathway. pathways, such as the PI3K/AKT/mTOR pathway, suggesting therapeutic potential for the treatment of prostate cancer	Nunes et al.
Anti-inflammatory effect	IL-1β-activated human synoviocytes	IL-1β-activated arthritic synoviocytes were treated with varying concentrations of *F. platyphylla* extracts and compounds. Negative control (untreated) was likely included for comparison	Concentrations of *F. platyphylla* extracts and isolated compounds ranging from 3 to 100 μg/mL	Root extract of *F. platyphylla* inhibited IL-6 release in arthritic synoviocytes	Salgado et al.
Antifungal activity	The fungal strains used were *C. tropicalis*, *C. guilliermondii*, *C. albicans*, and *C. krusei*	Negative control group: Dimethyl sulfoxide (DMSO); Positive control group: Ketoconazole at a concentration of 50 μg mL^–1^; *F. platyphylla* floral extract group at different concentrations	*In vitro:* 500; 250; 125; 62.5; 31.25 e 15.62 mg/mL diluted in DMSO	*F. platyphylla* floral extract showed antifungal activity at high concentrations	Menezes-Filho, Porfiro e Castro
Antifungal and cytotoxicity assay	Fungal strains of *C. albicans*, *C. krusei*, *C. tropicalis*, and *C. guilliermondii* were used	Antifungal assay: hydroethanolic solution (negative control) and ketoconazole (50 μg/mL, positive). In the cytotoxic assay: DMSO with seawater (control negative) and potassium dichromate with seawater (positive control). Extract was tested at various concentrations	*In vitro:* 100; 50; 25 e 12.5 mg/mL diluted in hydroethanolic solution (70%) for the antifungal assay	*F. platyphylla* extract inhibited *Candida* with greater activity in *C. krusei*; low toxicity in cytotoxicity test	Menezes-Filho
			1.000; 500; 100; 50; 25 e 1 μg/mL diluted in hydroethanolic solution (35%) for the lethality assay		
*In síilico:* electrochemical analysis of brachydins	Glassy carbon electrodes in buffered solution	Fractions 14, 15, and 16 of the extract were analyzed, which contained compounds called brachydins A, B and C, respectively.	The brachydin stock solutions brachydin A, brachydin B and brachydin C were prepared at 0.300 mmol/L in methanol	Brachydin A exhibited high oxidation at high pH, which affected its antioxidant capacity and redox behavior	Nascimento et al.
Cytotoxic activity	Panel of commercial human cell lines, four tumoral (U-251-glioblastoma, NCI-H460-lung, PC-3-prostate, and HT-29-colorectal), and one nontumorous (HaCat-keratinocyte)	(Doxorubicin hydrochloride—control group)	*In vitro:* concentrations of 1.6, 3.12, 6.25, 12.5, 25, 50, and 100 μg/mL in triplicate	Brachydins E and F exhibited cytotoxic effects in almost all cell lines tested, with selectivity for the PC-3 lines	Lima et al.
		Hydromethanolic subfraction group of the extract of *F. platyphylla* brachydin E and brachydin F			
Prostate cancer cells	Three-dimensional tumor models created from metastatic prostate cells of the DU145 lineage isolated from a metastatic brain site and reproduced in oncological models of the tumor spheroid type	Negative control RPMI 1640	*In vitro:* Brachydin A solutions were prepared in DMSO to reach final concentrations of 10, 20, 40, 60, 80, and 100 μM	Brachydin A showed cytotoxicity and reduced invasiveness and migration in tumor spheroids	Ribeiro et al.
		Solvent control DMSO (1%)			
		Positive control group: Docetaxel (50 μM)			
		Brachydin A group: Spheroids were treated with different concentrations of brachydin A			
Antitumor effects	DU145 metastatic prostate cancer cells in 2D and 3D models	PBS (Negative control): Solvent group with 0.25% DMSO; Brachydin B groups in 2D model: 0.24, 0.75, 0.96, 1.50, 3.84, 6.00, 15.36, 24.00, and 30.72 μM.	Brachydin B solutions were prepared in DMSO to reach final concentrations of 0.24, 0.75, 0.96, 1.50, 3.84, 6.00, 15.36, 24.00, and 30.72 μM of brachydin B for 2D culture and concentrations of 5, 10, 20, 30, 40, 50, and 60 μM for 3D culture	Brachydin B reduced cell viability in 2D model, inhibited spheroid growth and migration in 3D model.	Sperloni et al.
		Brachydin B groups in 3D model: 5, 10, 20, 30, 40, 50, and 60 μM.			
Antiparasitic and Cytotoxic effects	Nanotechnological formulation of brachydin A for antileishmania and cytotoxicity effects	Polymer F127 m/v 0.125%, 0.25% and 0.5% (negative control) Brachydin A Brachydin A-loaded F127 m/v 0,125%	Brachydin-loaded micelles were prepared in concentrations of 0.5%, 0.25%, or 0.125% (w/v) of F127, with a fixed brachydin A concentration of 500 μg/mL	The nanotechnological formulation of Brachydin A presented promising leishmanicidal activity, selective toxicity, and a favorable safety profile.	Costa et al.
		Brachydin A-loaded F127 m/v 0.25%			
		Brachydin A-loaded F127 m/v 0,50%			
Antiparasitic effect	*In vitro* exposure for 48 h (promastigotes and intracellular amastigotes); cytotoxicity in RAW 264.7 macrophages	ZNP-DCMF, DCMF, Blank ZNP, Pentamidine, Negative control (1% DMSO)	0.06–125 μg/mL; CC_50_ > 500 μg/mL	ZNP-DCMF: IC_50_ = 36.33 μg/mL (promastigotes), 0.72 μg/mL (amastigotes), SI = 13.77 and 694.44, respectively. DCMF: IC_50_ = 253.1 μg/mL (promastigotes), 6.96 μg/mL (amastigotes), SI = 1.98 and 71.84. Both formulations showed low cytotoxicity (CC_50_ > 500 μg/mL)	Neves et al.

a Prepared by the authors from the PubMed, Scielo, and Google Scholar databases. **Legend**: 15-LOX—15-Lipoxygenase; ACP02—Human gastric adenocarcinoma cell line; BCL-XL—B-cell lymphoma-extra large (antiapoptotic gene); BIRC5—Baculoviral IAP Repeat Containing 5 (Survivin gene); CC_50_—Cytotoxic Concentration 50%; DCMF—Dichloromethane fraction of extract; DMSO—Dimethyl Sulfoxide; DXR—Doxorubicin; EO—Essential Oil; F127—Pluronic F127 copolymer; FLAB-DCM—Dichloromethane extract of F. platyphylla; FLAB-Et—Ethanol extract of F. platyphylla; FP—Fridericia platyphylla; GAS—Primary human gastric cells; HT-29—Human colorectal adenocarcinoma cell line; IC_50_—Inhibitory Concentration 50%; IL-1β—Interleukin 1 β; IL-6—Interleukin 6; MET—Mesenchymal Epithelial Transition factor gene; MTT—3-(4,5-dimethylthiazol-2-yl)-2,5-diphenyltetrazolium bromide; NHA—Normal Human Astrocytes; PBS—Phosphate Buffered Saline; PC-3—Human prostate cancer cell line; PI3K/AKT/mTOR—Phosphoinositide 3-kinase/Protein kinase B/Mechanistic target of rapamycin pathway; RAW 264.7—Murine macrophage cell line; SI—Selectivity Index; TMZ—Temozolomide; U-251—Human glioblastoma cell line; ZNP—Zein nanoparticles; ZNP-DCMF—Zein nanoparticles containing dichloromethane fraction.

**Table 3:** Experimental Design, Dosing, and Outcomes of *In Vivo* Studies with *F. platyphylla* ^a^

experimental model	administration form	model system specifications	groups	*In vivo* monitoring	dose or concentration administered *in vivo*/*in vitro*	main findings	references
Effect on mice infected with *T. cruzi*	Oral gavage (once a day) for consecutive days	BALB/c mice (female, 6–8 weeks)	Control, Benznidazole, Amphotericin B, brachydin A, brachydin B, and brachydin C (*n* = 6)	30 days postinfection	100 mg/kg (*in vivo*); 1.23–100 μg/mL (*in vitro*)	Brachydin B and C reduced parasitemia in *T. cruzi*	Rocha et al.
Antiulcer effect in rats (induced ulcers)	Single dose, oral gavage	Wistar rats (male, 6–8 weeks)	Control group: (saline 10 mL/kg)	Days 0, 7, and 14 postinduction	10, 30, 100 e 300 mg/kg	HEAb demonstrated antiulcer activity comparable to that of lansoprazole	Rocha et al.
			Lansoprazole group: (60 mg/kg)				
			HEAb groups (10, 30, 100, and 300 mg/kg)				
Nociception test in adult mice	Oral gavage, once a day, single dose	Adult male Swiss mice, 20–35 g, *n* = 8	Saline solution 10 mL/kg	Immediate postdose	10, 30 ou 100 mg/kg	DEAB reduced pain in mice in the formalin test	Rodrigues et al.
			DEAB fraction 30 mg/kg				
			Morphine 2.5 mg/kg				
Effect against *L. amazonensis*	Oral gavage, once a day, for *3 weeks*	Female BALB/c mice (4–8 weeks); macrophages from BALB/c	Control, Amphotericin B and brachydin B	3 weeks after treatment	*In vitro:* dose de 0.25 a 20 μM	Brachydin B was effective against *L. amazonensis* promastigotes with no significant toxicity	Rocha et al.
					*In vivo*: maximum of 2 μM		
DCM fraction in microemulsion	Single-dose injection	*In vivo:* Evaluation of cytotoxicity of healthy larvae of the species *Tenebrio molitor* weighing approximately 100–200 mg	Control group with 1% DMSO, DCM fraction (10 μL), and ME3 groups with concentrations of 1 μg/kg, 5 μg/kg, 10 μg/kg, 50 μg/kg, and 100 μg/kg. For the *in vitro* test, a solution containing DCM fraction (LC) was compared with microemulsion groups containing DCM at concentrations of 3 and 5%	The viability of the larvae was monitored 24 h after the injections were administered, assessing the absence of movement over 7 days	*In vivo:* were administered 10 μL doses of DCM fraction; microemulsion groups received 1–100 μg/mL	The microemulsion demonstrated high stability and a safe release of DCM in larvae	Nascimento et al.
		*In vitro:* ME of DCM fraction were formulated at concentrations of 3% and 5% (ME3 and ME5)			*In vitro:* Tests used a saturated DCM fraction solution compared with microemulsions at 3 and 5% DCM		

a **S**ource: Prepared by the authors from the PubMed, Scielo, and Google Scholar databases. Legend: BALB/c—Albino laboratory-bred mouse strain (Bagg Albino Laboratory-Bred); DCM—Dichloromethane; DEAB—Dichloromethane extract of F. platyphylla; F. platyphylla—Fridericia platyphylla; HEAb—Hydroethanolic extract of F. platyphylla; LC—Liquid Chromatography (or DCM solution, context-dependent); ME3—Microemulsion 3%; ME5—Microemulsion 5%; Tenebrio molitor—Larvae used as *in vivo* toxicity model organism; Wistar—A common strain of albino rats used in laboratory research.

The chemical structures of the isolated compounds listed in Table 1 are shown in Figure 1, allowing for easier visualization and identification of each described substance.

**Figure 1:**
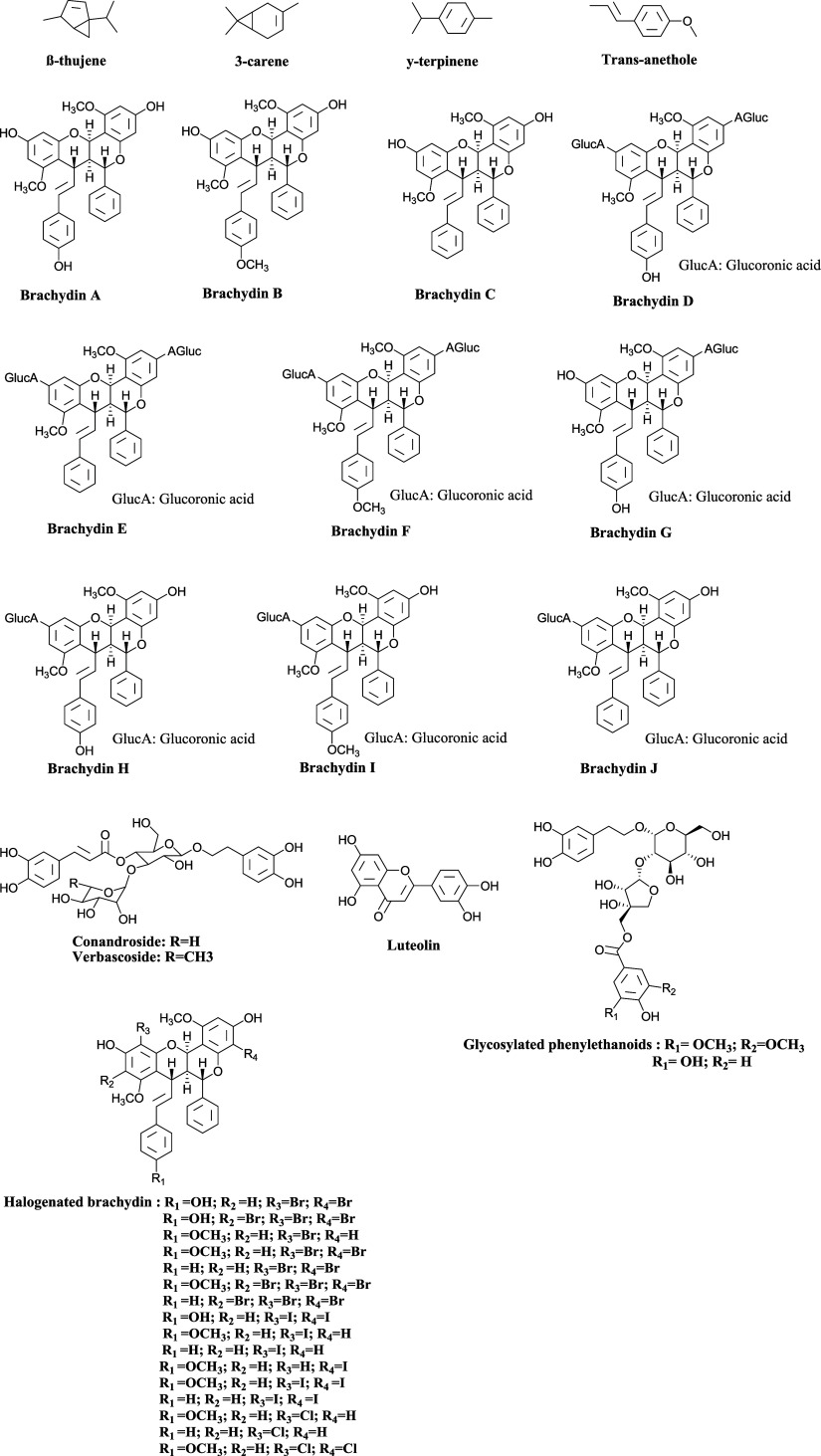
Structure of identified compounds from *F. platyphylla* or its synonym *A. brachypoda*.

Figure 2 shows the temporal evolution of the studies published using the crude extract and/or phytochemical compounds isolated from the species *F. platyphylla* in different experimental models. It shows that most of the publications of studies with this species were between 2019 and 2021.

**Figure 2:**
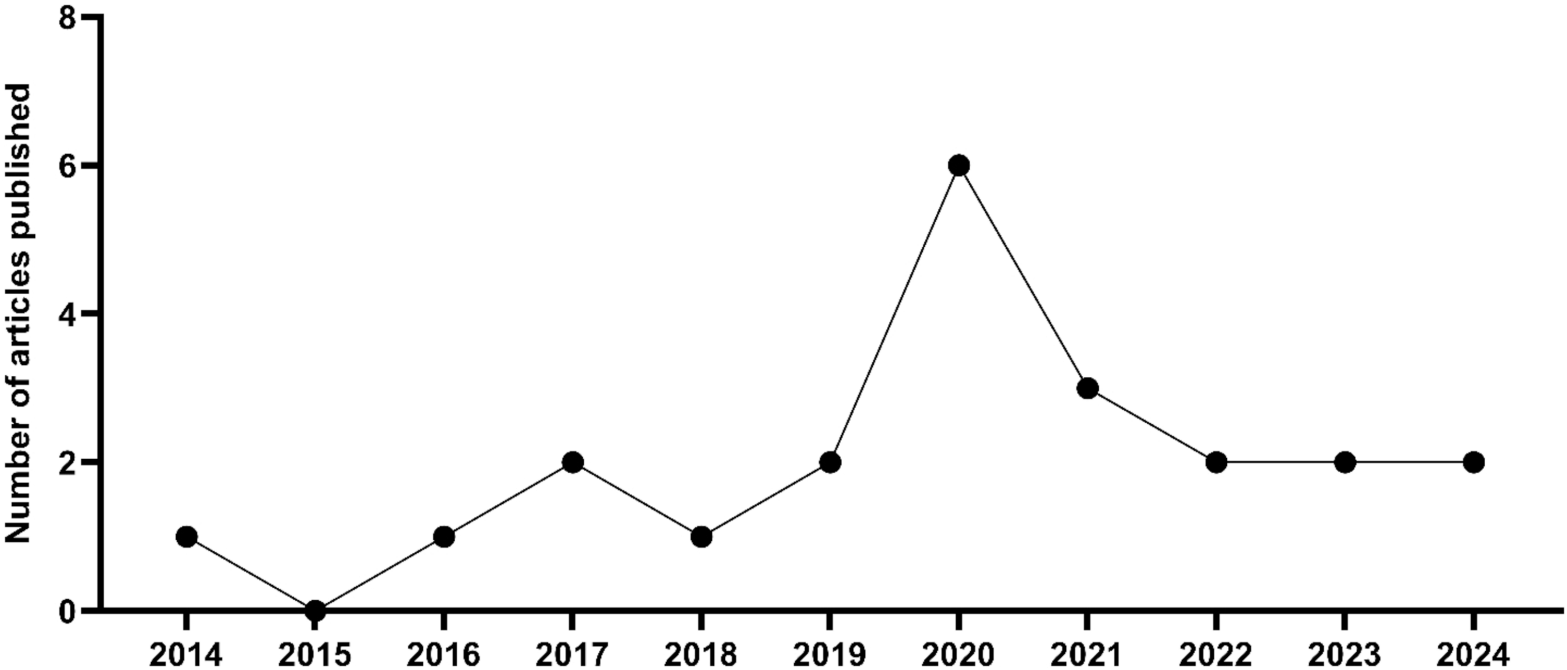
Temporal evolution of articles published with the species *F. platyphylla* or its synonym *A. brachypoda* (2014–2024). **Source**: Prepared by the authors from the PubMed, Scielo and Google Scholar databases.

Figure 3 illustrates the pharmacological effects observed in response to different doses of the crude extract or isolated phytochemical compounds from *F. platyphylla*. The figure highlights dose-dependent relationships, supporting the potential therapeutic relevance of both the crude extract and its active constituents.

**Figure 3:**
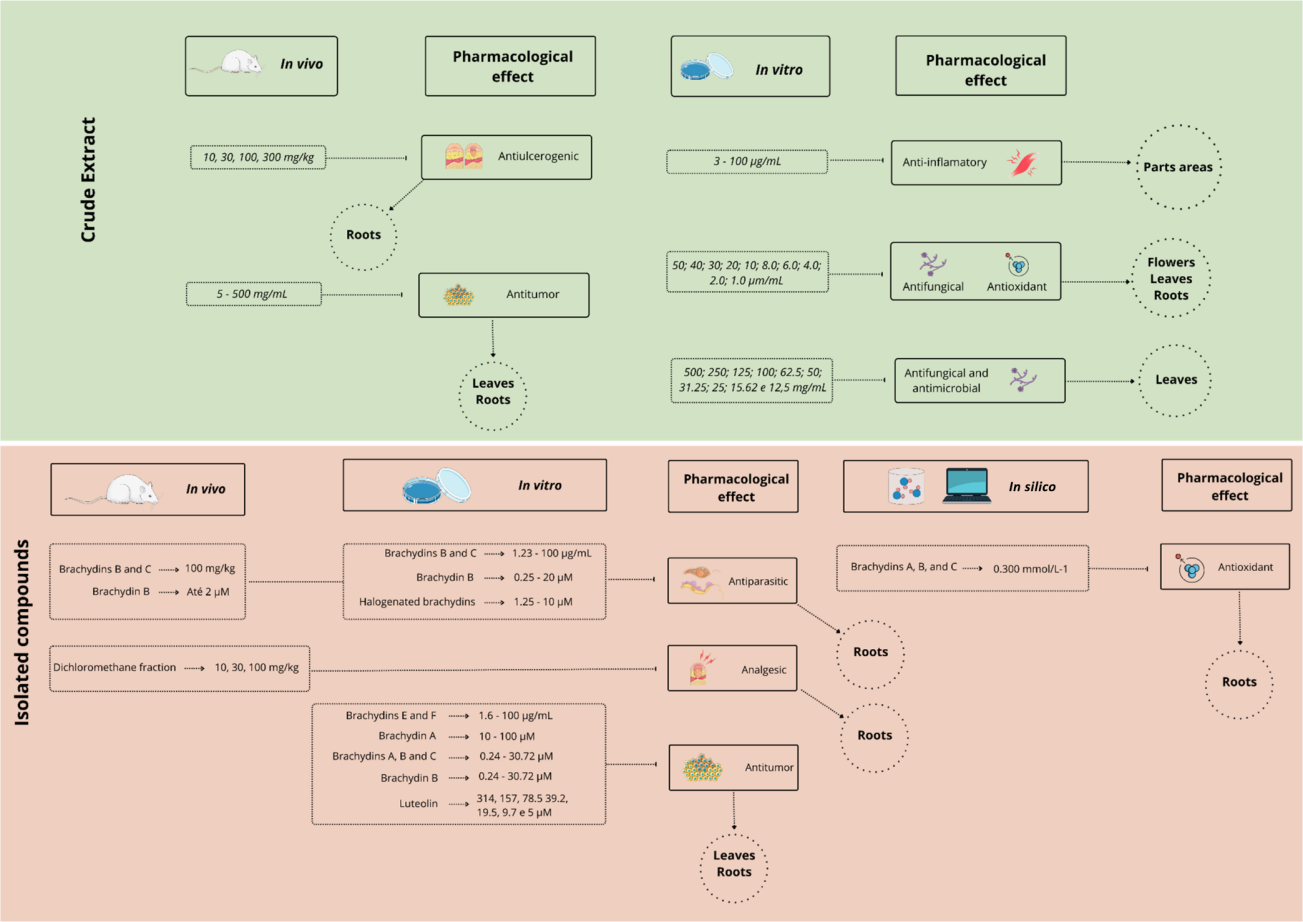
Pharmacological effects related to the dose of crude extract or phytochemical compounds isolated from *F. platyphylla*. **Source**: Prepared by the authors based on the studies included in this review. This Figure was created using Servier Medical Art (https://smart.servier.com/), licensed under CC BY 4.0 (https://creativecommons.org/licenses/by/4.0/).

Figure 4A,B illustrate the diverse effects of *F. platyphylla* across *in vitro*, *in vivo*, and *in silico* models.

**Figure 4:**
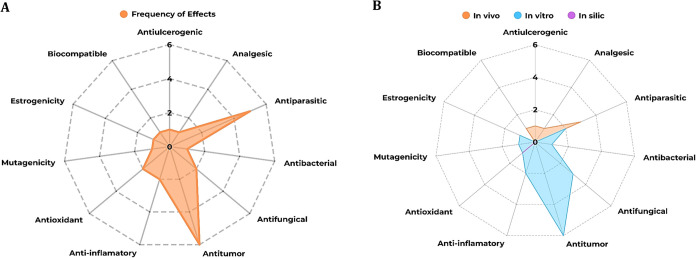
Effects related to the use of *F. platyphylla* or its synonym *A.* *brachypoda* *in vitro, in vivo*, and *in silico* models. Summary of pharmacological data related to *F. platyphylla*. (A) Frequency of pharmacological effects attributed to the crude extract or bioactive compounds. (B) Frequency distribution of study types investigating these effects, categorized *as in vitro, in vivo*, and *in silico*. **Source**: Prepared by the authors based on the studies included in this review.

Figure 5 illustrates the proposed mechanism of action and pharmacological activities of the bioactive compounds isolated from *F. platyphylla*. The figure integrates experimental evidence from *in vitro* and *in vivo* assays, highlighting key effects such as antiparasitic, antitumoral, anti-inflammatory, antioxidant, antimicrobial, and analgesic actions. These effects are primarily attributed to brachydins, which modulate cellular targets including efflux pumps, inflammatory mediators (such as IL-6), reactive oxygen species (ROS), and apoptotic pathways. The schematic representation provides a comprehensive overview of how these compounds interact with molecular targets, supporting their therapeutic potential in different experimental models.

**Figure 5:**
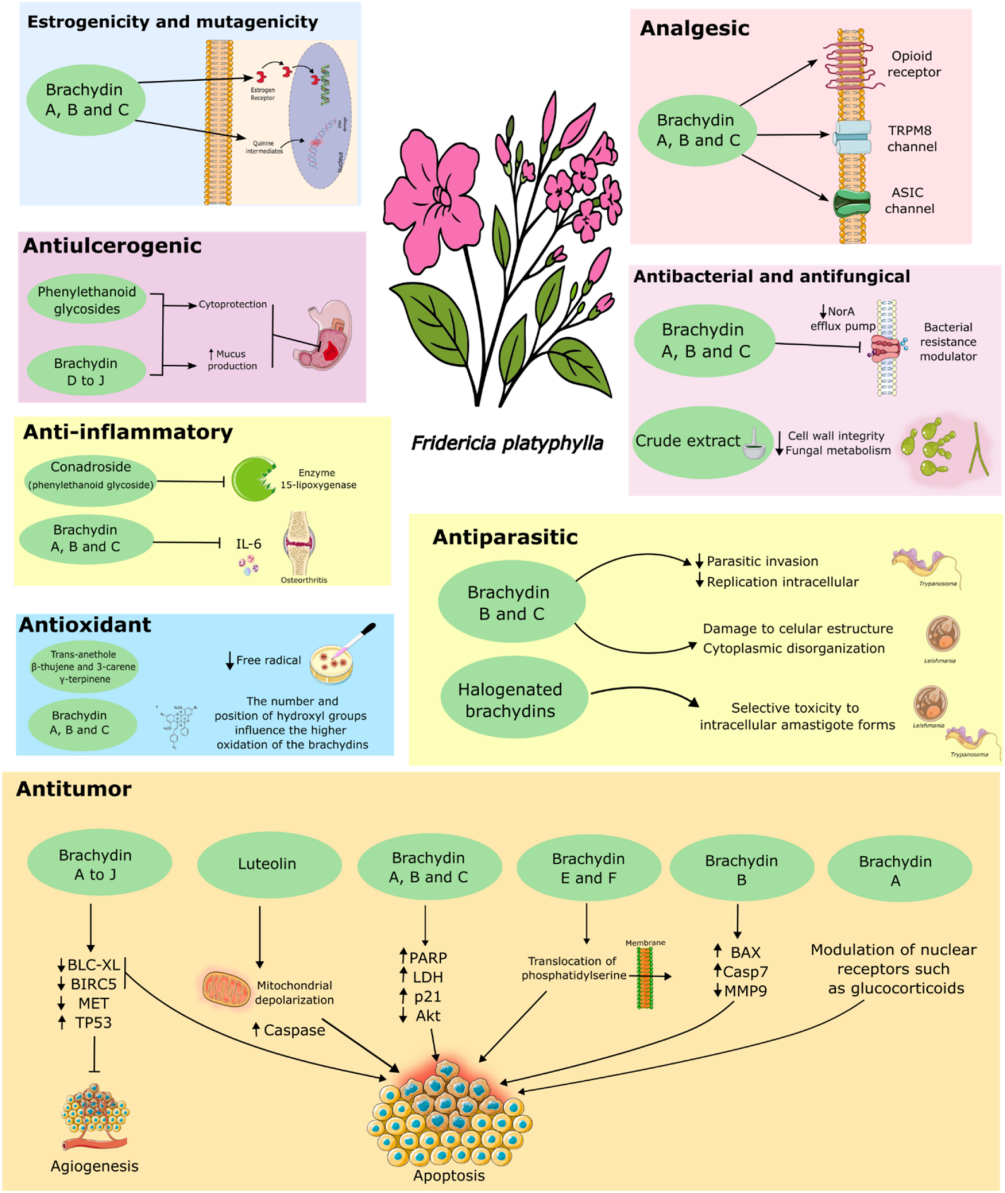
Mechanism of action and pharmacological activies of bioactive compounds from *F. platyphylla*. Representative image of the main results obtained from the use of the extract and/or compounds isolated from the species *F. platyphylla* in different experimental models. **Source**: Prepared by the authors based on the studies included in this review. This Figure was created using Servier Medical Art (https://smart.servier.com/), licensed under CC BY 4.0 (https://creativecommons.org/licenses/by/4.0/).

Figure 6 illustrates the advanced drug delivery systems developed using Fridericia platyphylla and its bioactive compounds. The diagram highlights three main nanotechnological approaches: microemulsions, polymeric micelles, and zein nanoparticles, each designed to improve solubility, stability, and bioavailability. These delivery platforms have demonstrated enhanced encapsulation efficiency, sustained release profiles, low cytotoxicity, and high selectivity in *in vitro* and *in vivo* models

**Figure 6:**
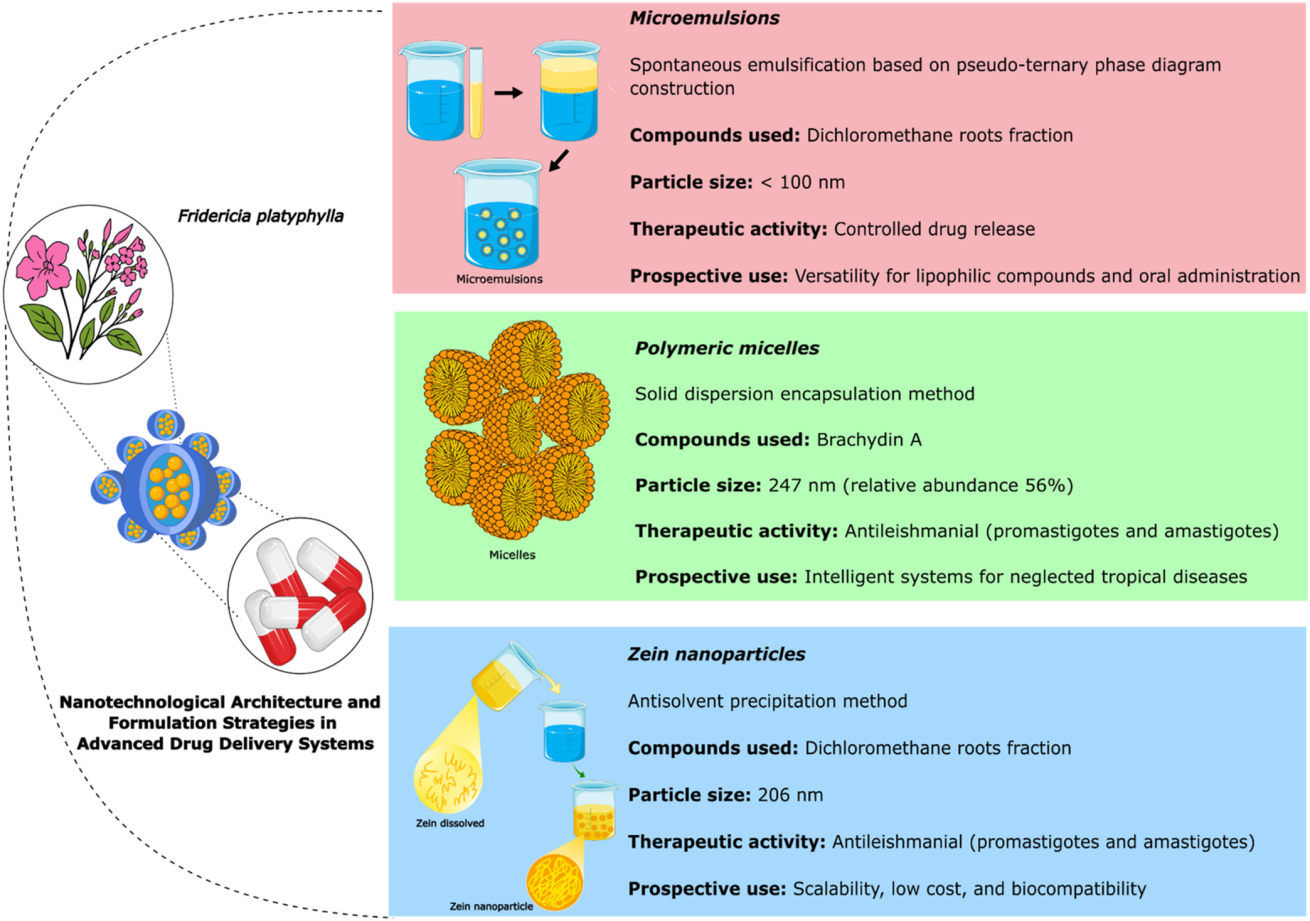
Advanced Drug Delivery Systems Based on *F. platyphylla*: Microemulsions, Polymeric Micelles, and Zein Nanoparticles Representative image of the main results obtained in advanced drug delivery systems based on *F. platyphylla*. **Source**: Prepared by the authors based on the studies included in this review. This Figure was created using Servier Medical Art (https://smart.servier.com/), licensed under CC BY 4.0 (https://creativecommons.org/licenses/by/4.0/).

## 3 Discussion

The growing phytochemical and pharmacological exploration of *F. platyphylla* underscores its promise as a source of bioactive compounds with diverse therapeutic potential. The data compiled in this review consolidate evidence for antioxidant, anti-inflammatory, antiparasitic, antitumor, antinociceptive, and antimicrobial effects associated with both crude extracts and isolated compounds, particularly dimeric flavonoids such as brachydins (A–F), luteolin, and cuneatin. These findings suggest that structural diversity and chemical complexity significantly influence pharmacological activity, modulated by electron-donating and withdrawing substituents, medium pH, lipophilic properties, and controlled-release formulations such as microemulsions.

Notably, both *in vitro* and *in vivo* studies demonstrate not only therapeutic efficacy but also selectivity and low cytotoxicity in nontumor cells, with well-characterized molecular mechanisms of action. These include inhibition of inflammatory pathways (*e.g.*, LOX, IL-6), mitochondrial-mediated apoptosis, ion channel modulation related to nociception, and interference with cell proliferation signaling (*e.g.*, pAKT pathway). Standardization of extracts, rigorous structural characterization, and the use of robust analytical methodologies—such as HPLC, UHPLC-MS/MS, NMR, and ECD—strengthen the reliability of these findings and provide a solid scientific foundation for translational research. This is particularly relevant when coupled with nanotechnological strategies aimed at improving bioavailability, stability, and therapeutic safety.

Additionally, a new visual summary (Figure 5) was inserted to illustrate the mechanisms of action and pharmacological activities of the major bioactive compounds, facilitating a clearer understanding of their biological relevance.

The following sections discuss the main pharmacological activities reported for crude extracts and isolated bioactive compounds of *F. platyphylla* as documented in the studies included in this review.

### 3.1 Biological Potential, Antioxidant Effect and Possible Pharmacological Mechanisms

The search for the biological effects of extracts and bioactive compounds isolated from *F. platyphylla*, in different experimental models, has been a constant concern of the scientific community, which strives to produce studies that explain the pharmacological mechanisms associated with this species.^19,20,22^ Interestingly, between 2019 and 2021, the number of published studies related to the species F. platyphylla increased significantly, reflecting the technological potential of the species for potential pharmaceutical, nutraceutical, and/or cosmetic applications of its extracts and isolated compounds. It is worth mentioning that in 2020, there was a peak in the number of published studies (*n* = 6), with a subsequent reduction in subsequent years (Figure 2). The growth in the number of publications related to the species reflects the evaluation of its anti-inflammatory, antioxidant, antimicrobial, and/or antiparasitic potential.

It is noteworthy that several pharmacological applications were observed due to the different characteristics of the extracts and their fractions obtained in the studies included in this review.^8,9,23^ In a survey conducted by Nascimento et al., the diversity of pharmacological applications related to the characteristics of the extracts, particularly the fractions of the F. platyphylla plant, was highlighted. The research focused on the electrochemical investigation of three unusual dimeric flavonoids, Brachydins A, B and C (Figure 1), which were isolated and identified for the first time by the research group. To isolate brachydins, *F. platyphylla* roots were subjected to exhaustive percolation extraction using a mixture of ethanol and water (7:3). The crude hydroethanolic extract obtained was then subjected to a liquid/liquid partition using dichloromethane (CH_2_Cl_2_) and a water/methanol solution (7:3). The resulting fraction was analyzed by high-performance liquid chromatography with photodiode array detection (HPLC-PDA) at λ = 254 nm. Cyclic voltammetry (CV) and differential pulse voltammetry (DPV) revealed that brachydin A is more easily oxidized, presenting an oxidation peak at +0.48 V, compared to brachydins B (+0.71 V) and C (+0.57 V) due to the presence of an hydroxyl group (−OH) in the D ring. The research also highlights the significant influence of the substituent in the D ring and the pH of the environment on the antioxidant capacity and redox behavior of the brachydins. With increasing pH, the oxidation potentials of the three brachydins shift to less positive values, confirming a direct relationship between pH and the facilitation of flavonoid oxidation, where oxidation is more pronounced for brachydin A due to its chemical structure.^18^ Another important discussion was the influence of chemical substituents attached to the aromatic rings of flavonoids. The results indicate that electron-attracting groups, such as nitro (–NO_2_) and carbonyl (C=O) groups, tend to hinder oxidation, while electron-donating groups, such as methoxyl (−OCH_3_) and hydroxyl (−OH), can facilitate electron loss, reflecting how chemical substitution can impact the redox behavior of flavonoids. Furthermore, everything revealed that the hydroxyl groups of ring B of flavonoids did not undergo oxidation. This is due to the lack of resonance between rings B and C, as well as the absence of a double bond and electronegative groups, which justify the electrochemical inactivity of ring B.^24^

Phenolic compounds, especially flavonoids, are known for their antioxidant properties, which are closely linked to the presence of hydroxyl groups.^24^ In previous studies, such as that carried out by Gomes et al.,^25^ it was observed that the addition of more hydroxyl groups to flavones and 2-styrylchromones led to a decrease in the anodic peak potential, indicating that these compounds became progressively more susceptible to oxidation. However, the oxidation dynamics of brachydins—a specific type of phenolic dimers—reveal a different behavior. Despite also containing hydroxyl groups, the molecular structure of brachydins modifies their electrochemical properties. Specifically, the −OH group located in ring B is not in resonance with ring C. The lack of resonance implies that the electron density is not efficiently shared between the rings, resulting in greater difficulty in oxidizing the −OH group of ring B, particularly when compared to compounds with rings that present effective resonance between hydroxyl groups and aryl structures.^24^

### 3.2 Potential Antiparasitic Treatment

Rocha et al. evaluated the efficacy of nonpolar fraction of an aqueous ethanol extract of the roots of *F. platyphylla* against *T. cruzi*, the parasite responsible for Chagas disease. The extract was subjected to liquid–liquid partitioning with dicloromethane (CH_2_Cl_2_) and water:methanol (H_2_O-MeOH, 7:3) and then fractionated by medium-pressure liquid chromatography (MPLC) using a Zeoprep C18 column. This resulted in 235 fractions, among these, Brachydins A, B, and C were identified. Their structures were elucidated using Ultraviolet spectroscopy (UV), Nuclear Magnetic Resonance (NMR), and High-Resolution Mass Spectrometry (HRMS) analysis, as well as by chemical derivatization. Brachydin A showed no activity against trypomastigotes *T. cruzi* (IC_50_ > 20 μM), while Brachydins B and C exhibited selective activity with inhibitory concentration (IC_50_) values of 5.3 and 6.6 μM, respectively. Both inhibited the parasite’s invasion and intracellular development, comparable to benznidazole (IC_50_ 11.3 μM), reference compound. Against intracellular amastigotes of *T. cruzi*, Brachydins B and C demonstrated IC_50_ values of 6.0 μM and 6.8 μM. In comparison, the reference drug benznidazole exhibited an IC_50_ value of 14.0 μM in the same assay. This indicates that Brachydins B and C could potentially inhibit the parasite’s intracellular development effectively, making them promising candidates for the development of new anti-*T. cruzi* drugs.^11^

In 2019, the same group evaluated the antileishmanial activity of flavonoids Brachydins A, B and C against promastigotes and amastigotes of three *Leishmania* species (*L. amazonensis*, *Leishmania infantum* and *Leishmania braziliensis*) using the viability assay based on the metabolism of Alamar Blue. Similar to *T. cruzi* activity, Brachydin A also demonstrated no significant activity against *Leishmania* species, with an IC_50_ greater than 20 μM. While Brachydin B and C demonstrated activity against all three tested species of *Leishmania* promastigotes, it was Brachydin B (*L. amazonensis*, IC_50_ = 9.16 μM; *L. braziliensis*, IC_50_ = 7.05 μM; *L. infantum*, IC_50_ = 12.90 μM) that stood out significantly. Brachydin B not only exhibited superior potency compared to the other compounds, but it was also the most effective against intracellular amastigotes of *L. amazonensis*, with an IC_50_ value of approximately 2.20 μM. Its ability to inhibit the proliferation of intracellular forms of the parasite without causing toxicity to host cells makes Brachydin B a promising candidate for the development of new treatments for leishmaniasis. The positive control used was amphotericin B, which presented an IC_50_ of 0.14 μM against *L. amazonensis*, 0.11 μM against *L. braziliensis* and 0.05 μM against *L. infantum*. Notably, Brachydin B showed a significant reduction in lesion size in an experimental model of cutaneous leishmaniasis.^23^

The studies demonstrate that, although Brachydin A does not present significant activity against *T. cruzi* and *Leishmania* spp, Brachydins B and C exhibit promising potencies. The presence of functional groups and the structural configuration of Brachydins, such as methoxylated groups and substituents that promote greater lipophilicity, are fundamental for their ability to penetrate the cell membranes of parasites and enhance their antiprotozoal activity. Brachydin B, in particular, demonstrated remarkable selectivity and efficacy in inhibiting the proliferation of intracellular amastigotes, and also stands out for not causing toxicity to host cells.^11,17^

The study carried out by Neuenshwander et al. described how the halogenation process can increase the biological activity of phenolic compounds present in the dichloromethane (CH_2_Cl_2_) fraction of *F. platyphylla* root extract against *L. amazonensis* and *T. cruzi*. The halogenation reactions used eco-friendly conditions with sodium bromide (NaBr), sodium iodide (NaI), and sodium chloride (NaCl), monitored by Ultra-High Performance Liquid Chromatography with Photodiode Array, Evaporative Light Scattering, and Mass Spectrometry (UHPLC-PDA-ELSD-MS). The halogenated derivatives were isolated *via* semipreparative High-Performance Liquid Chromatography (HPLC-UV) and characterized by Nuclear Magnetic Resonance (NMR) and High-Resolution Mass Spectrometry (HR-MS). All the 16 halogenated derivatives were evaluated for their antiparasitic activities against the parasites *L. amazonensis* and *T. cruzi*. Compounds 10 (IC_50_ = 1.0 μM) and 18 (IC_50_ = 1.2 μM) exhibited particularly strong selective antiparasitic activity against *L. amazonensis*. In contrast, compounds 8, 14, 17, and 18 demonstrated significant efficacy against *T. cruzi*, with IC_50_ values ranging from 1.4 to 1.6 μM. When compared to the positive control, amphotericin B, which has an IC_50_ of 0.2 μM, these compounds show promising potential as effective treatments for these parasitic infections. The study demonstrated that the introduction of halogens, such as bromine and iodine, in specific positions of these molecules contribute significantly to the expansion of antiparasitic efficacy, highlighting the potential of these halogenated derivatives as promising therapeutic agents against these parasitic infections.^19^

In parallel, a study evaluated the antiparasitic activity of brachydin A after its encapsulation into F127 polymeric micelles (LF-B500: Brachydin A-loaded F127 m/v 0,125%; Brachydin A-loaded F127 m/v 0,25%; and Brachydin A-loaded F127 m/v 0,50%), which exhibited significant activity against *L. amazonensis* promastigotes, with an IC_50_ of 16.06 μg/mL for the LF-B500 formulation.^26^ Simultaneously, the formulation showed low cytotoxicity against murine RAW 264.7 macrophages, with a 50% cytotoxic concentration (CC_50_) of 171 μg/mL. The difference between the IC_50_ for the parasite and the CC_50_ for the host cells resulted in a high selectivity index (SI = 10.64) for promastigotes compared to RAW cells, suggesting that the formulation is more toxic to the parasite than to host cells - an essential factor in the development of safe and effective treatments for leishmaniasis.^26^

Neves et al. investigated the antileishmanial potential of the DCMF (roots), which are known to be enriched in the dimeric flavonoids Brachydins A, B, and C. The crude extract was obtained by percolation with an ethanol/water mixture (7:3) and subjected to liquid–liquid partitioning to isolate the DCMF.^27^ Chemical profiling using HPLC-PDA and LC-MS confirmed the predominance of brachydins in the fraction. To enhance the solubility, bioavailability, and biological performance of these compounds, DCMF was incorporated into zein nanoparticles (ZNP-DCMF) *via* antisolvent precipitation in the presence of Pluronic F-68.^27^ Among the concentrations tested (0.5 to 10 mg/mL), the formulation containing 0.5 mg/mL of DCMF showed the most outstanding colloidal stability and was selected for further assays. The physicochemical properties of ZNP-DCMF were evaluated through dynamic light scattering (DLS), ζ-potential, and atomic force microscopy (AFM), revealing particles with an average diameter of 206 nm, a polydispersity index below 0.2, and spherical shape.^27^ Encapsulation was further confirmed by Fourier-transform infrared spectroscopy (FTIR), which evidenced physical interactions through the suppression of characteristic DCMF signals, and by differential scanning calorimetry (DSC), which showed thermal shifts indicative of matrix–flavonoid integration.^27^ The formulation achieved an encapsulation efficiency above 94% and maintained structural stability for at least 49 days at room temperature. Biological evaluation of ZNP-DCMF against promastigote and intracellular amastigote forms of *L. amazonensis* revealed an IC_50_ of 36.33 and 0.72 μg/mL, respectively—the latter significantly outperforming the free DCMF (IC_50_ = 6.96 μg/mL). In cytotoxicity assays with RAW 264.7 murine macrophages, the formulation demonstrated low toxicity (CC_50_ > 500 μg/mL), resulting in a high SI (694.44). Overall, these results highlight that nanoencapsulation markedly improves the physicochemical and pharmacological attributes of DCMF, underscoring the promise of ZNP-DCMF as a candidate for innovative antileishmanial therapies.^27^

### 3.3 Potential Antinociceptive and Anti-Inflammatory

Rocha et al. investigated the mechanisms of action of the bioactive compounds of the hydroethanolic extract of the root of *F. platyphylla*. obtained by method of percolation with ethanol and water (70% v/v). The characterization of the compounds included UV spectroscopy, nuclear magnetic resonance (NMR), and high-resolution mass spectrometry (HRMS), resulting in the identification of seven glycosylated dimeric flavonoids and two unprecedented derivatives of phenylethylamine glycosides in literature. The absolute configuration of the isolated dimeric flavonoids was determined using electronic circular dichroism (ECD) spectroscopy. The gastroprotective effects were tested in male Wistar rats in several models of gastric ulcer, showing a significant reduction in gastric damage at a dose of 300 mg/kg, similar to lansoprazole (60 mg/kg). The metabolization of the compounds suggests that glycosides and aglycones may be involved in the observed effects. Appropriate controls, including carbenoxolone (100 mg/kg), confirmed that the extract did not affect motor coordination, validating that its gastroprotective effects were not due to sedation. The results highlight its potential as a gastroprotective agent and corroborate its use in folk medicine.^4^

In addition, the study examined the mechanisms of action of bioactive compounds present in the dichloromethane fraction (DEAB) from the roots of *F. platyphylla*.^20^ The DEAB fraction was obtained through liquid–liquid extraction, using dichloromethane as a solvent. The characterization of the compounds was performed by high-performance liquid chromatography (HPLC), which accurately identified three dimeric flavonoids known as brachydins A, B, and C. The antinociceptive activity of DEAB was tested in male Swiss mice using formalin and hot plate tests. The fraction demonstrated efficacy at doses ranging from 10 to 100 mg/kg, indicating its potential as an antinociceptive agent. The study also investigated the mechanisms of action, identifying the involvement of ion channels such as TRPV1 (transient receptor potential vanilloid 1), ASIC (acid-sensing ion channels), TRPM8 (transient receptor potential melastatin 8), and TRPA1 (transient receptor potential ankyrin 1) in pain modulation. Control tests were conducted, including groups treated with a vehicle and diazepam (2 mg/kg) as a positive control, which induced a sedative effect and allowed for the assessment of whether DEAB affected the motor coordination of the mice. Locomotor performance was evaluated using a rotarod apparatus, revealing that DEAB did not significantly influence the motor coordination of the animals, confirming that the antinociceptive effect was specific and not due to a sedative effect.^20^

*F. platyphylla* is associated with inflammatory process regulation in several pathologies, such as rheumatoid arthritis, atherosclerosis, and cancer.^20,28,29^ Research carried out by Bertanha et al. investigated the anti-inflammatory potential of *F. platyphylla* through the inhibition of lipoxygenase (LOX), an enzyme involved in inflammatory processes. The ethanolic extract from the aerial parts of the plant was subjected to microfractionation using high-performance liquid chromatography (HPLC-DAD) to identify bioactive compounds. The microfractions were collected and tested in *in vitro* assays to evaluate their ability to inhibit LOX. Additionally, the crude extract was analyzed by high-resolution mass spectrometry (HPLC-HRMS) to identify the components present, while the extract was purified through solid-phase chromatography and semipreparative HPLC. The isolated compound, identified as conandroside, exhibited LOX inhibitory activity with a IC_50_ of 7.8 μM, similar to quercetin (IC_50_ of 7.6 Mm). The study found no significant toxicity of conandroside in normal cells, suggesting its potential as a lipoxygenase inhibitor and its value in the search for new anti-inflammatory agents.^3^

Given the similarity in lipoxygenase (LOX) inhibitory activity between conandroside and quercetin, molecular docking simulations of the two compounds become essential for elucidating their interactions. Conandroside forms hydrogen bonds with His373 and Ile676, stabilizing the enzyme complex and inhibiting the enzyme’s activity. Its interaction with the Fe^3+^ ion modifies the redox cycle of LOX, while π-π and cationic interactions with Phe192 and Arg429 enhance its efficacy. Quercetin, although it has fewer hydrogen bonds, possesses a compact structure that promotes strong interactions with LOX, resulting in a similar inhibition profile. This understanding of the structure–activity relationship (SAR) is crucial for the development of new LOX inhibitors.^3^

On the other hand, Salgado et al. focused on the modulation of the levels of the pro-inflammatory cytokine IL-6 from the dichloromethane (DCMAB) and hydroethanolic (HEAB) fractions derived from the root extract of *F. platyphylla*. High-performance liquid chromatography with photodiode array detection (HPLC-PDA) was used to analyze the fractions, while the DCMAB was fractionated by medium-pressure liquid chromatography (MPLC), and the dimeric compounds brachydins A, B, and C were isolated using high-speed counter-current chromatography (HSCCC). For quantification, the fractions were analyzed by UHPLC-MS/MS, increasing the sensitivity in determining the bioactive compounds present. The Enzyme-Linked Immunosorbent Assay (ELISA) method was used to quantify anti-inflammatory activity. This assay measured the levels of the pro-inflammatory cytokine IL-6 in synoviocytes activated with IL-1β after incubation with extracts and isolated compounds. The results demonstrated that the DCMAB effectively inhibited the release of IL-6, while the HEAB showed no significant effect. The higher presence of brachydins B and C in the DCMAB is related to its anti-inflammatory potential, highlighting the role of these compounds in the traditional use of the plant to treat inflammatory conditions such as osteoarthritis.^16^

### 3.4 Potential Antitumor Agent

In the study conducted by Franco et al., luteolin, a flavonoid isolated from *F. platyphylla*, demonstrated significant antiproliferative activity against glioblastoma multiforme (GBM), one of the most aggressive types of brain tumors. The dried leaves of *F. platyphylla* were extracted by exhaustive percolation with 70% ethanol at room temperature, followed by liquid–liquid partitioning and isolation of luteolin from the ethyl acetate fraction using silica gel column chromatography. The compound was identified through ultraviolet (UV) spectroscopy, nuclear magnetic resonance (NMR) spectroscopy, and mass spectrometry (MS). The cytotoxic activity of luteolin was evaluated against a panel of tumor cell lines (U-251, NCI-ADR/RES, 786–0, NCI-H460, PC-3, HT-29) and nontumor cell lines (HaCaT and NHA) using the MTT assay (3-(4,5-dimethylthiazol-2-yl)-2,5-diphenyltetrazolium bromide), which measures mitochondrial metabolic activity as an indirect indicator of cell viability. After 48 h of exposure, luteolin exhibited potent antiproliferative effects, achieving a GI_50_ value of 6.6 μM for U-251 cells, significantly lower than that of the standard chemotherapeutic agent Temozolomide (TMZ), which presented a GI_50_ of 873 μM. Although the selectivity index (SI) of luteolin (0.98) did not reach the ideal threshold (≥2.0), it was substantially higher than that of TMZ (0.06), suggesting relatively greater selectivity of luteolin toward tumor cells. Moreover, luteolin demonstrated lower toxicity to nontumor cells compared to TMZ. Additional functional assays revealed that luteolin significantly inhibited the migration and colony formation of U-251 cells, reducing the number of colonies by 82% after 21 days of treatment. Mechanistic studies indicated that luteolin induced apoptosis in U-251 cells through mechanisms involving mitochondrial membrane depolarization, phosphatidylserine externalization, cleavage of PARP and caspase-9, and increased phosphorylation of ERK and H2AX proteins, suggesting mitochondrial-mediated apoptosis and DNA damage.^30^

Studies carried out with the extract of *F. platyphylla* roots demonstrated selectivity in tumor cells, inducing necrosis and altering the expression of genes related to apoptosis and the cell cycle.^22^ The researchers investigated the cytotoxic and antiproliferative effects of *F. platyphylla* roots on normal (GAS) and tumor cells (ACP02 and HepG2). The extract was prepared by exhaustive percolation using a mixture of ethanol and water in a 7:3 ratio, at room temperature, and was analyzed by liquid chromatography coupled to mass spectrometry (LC/MS), identifying the presence of dimeric flavonoids, known as brachydins (A to J), which have recognized pharmacological properties, including cytotoxic activity. The effects of the extract were measured by cell viability assays, such as MTT and neutral red. The effective concentrations (EC_50_) obtained indicated significant efficacy: 56.16 mg/mL for GAS cells, 43.68 mg/mL for ACP02 and 42.57 mg/mL for HepG2. Furthermore, the data suggested a significant decrease in nuclear division rates in tumor cells treated with the extract, although this decrease was not reflected in the cell proliferation curves, implying that although the extract causes cell death, this does not necessarily translate into short-term cell growth inhibition. This complexity may be due to underlying mechanisms, such as the induction of apoptosis or necrosis, which may not manifest in a linear manner in the cell growth curves.^22^

The cytotoxic effects of Brachydins A, B, and C, dimeric flavonoids isolated from the roots of *F. platyphylla*, were investigated in a study using prostate cancer PC-3 cells. The isolation and structural elucidation of the brachydins were detailed in the work by Rocha et al., where advanced techniques such as nuclear magnetic resonance (NMR) and high-resolution mass spectrometry (HRMS) were utilized. The structural differences among the three brachydins are subtle, with BrA containing a hydroxyl group, BrB having a methoxy group, and BrC consisting of a single hydrogen. The study assessed the cytotoxic effects of brachydins on the PC-3 cell line using concentrations ranging from 0.24 to 30.72 μM and employed three viability assays: MTT, neutral red, and LDH release. Results indicated that brachydins significantly decreased cell viability compared to the negative control (PBS), with brachydins B exhibiting the highest cytotoxicity. The IC_50_ values were determined as 23.41 μM for brachydins A, 4.28 μM for brachydins B, and 4.44 μM for brachydins C. Morphological analysis showed increased apoptosis induced by the brachydins, particularly with brachydins B at 6 μM, significantly reducing cell viability. Compared to the positive control (Docetaxel at 10 μM), brachydins demonstrated promising effects, especially brachydins B, implying its potential as an alternative or complementary cancer treatment. Furthermore, elevated levels of p21 protein were observed in cells treated with brachydins B and brachydins C, suggesting a potential G1 phase cell cycle arrest. Analysis of pAKT protein expression showed significant reductions following treatment with BrA and BrB, indicating that these brachydins impact cell proliferation pathways. Overall, these findings highlight brachydin B as an appealing candidate for future investigations in cancer therapies.^8^

The studies carried out on the flavonoids Brachydin A and Brachydin B illustrate the promising antitumor effects and the mechanisms of action in DU145 metastatic prostate cancer cells, emphasizing their potential therapeutic applications.^21,24^ Brachydin A demonstrates significant cytotoxic properties in tumor spheroids of DU145, with cytotoxicity observed in concentrations of 60 to 100 μM, as evidenced by acid phosphatase and resazurin assays. The antitumor activity of BrA has been attributed to mechanisms that include the promotion of DNA damage and the induction of parthanatos, a form of cell death mediated by the activation of poly­(ADP-ribose) polymerase (PARP). Furthermore, Brachydin A increases the activity of caspases and alters the levels of anti/pro-apoptotic markers, indicating their interference in the signaling pathways of cell survival, making tumor cells more susceptible to apoptose. Brachydin A also demonstrates effectiveness in inhibiting cellular migration and invasion, addressing a critical challenge in the management of metastatic spread of prostate cancer. Compared to the positive control Docetaxel (50 μM), which is a parent chemotherapeutic agent, or Brachydin A can offer a therapeutic alternative with lower toxicity potential for normal cells, promoting a safer and more effective approach in the treatment of the disease.^21^

Similarly, the study on the flavonoid Brachydin B in metastatic DU145 cells revealed promising antitumor and antimigratory effects. O Brachydin B exhibited cytotoxicity from 1.50 μM after 24 h in 2D cultures, as measured by MTT and resazurin assays. In 3D tumor spheroid models, cytotoxicity was observed in higher concentrations after prolonged periods. BrB also reduces clonogenicity in 2D cultures and reduces the volume of tumor spheroids. Furthermore, we inhibited cell migration at 6 μM in 2D monolayers and showed antimigratory effects in spheroids starting at 30 μM. These results highlight BrB as a potential therapy for metastatic prostate cancer, encouraging additional research.^24^ The details regarding the extraction, isolation, and identification of Brachydin A and B were previously reported by Rocha et al.

These compounds, Brachydin A and Brachydin B showed potential as therapeutic agents against metastatic prostate cancer. While Brachydin A can induce cell death through specific mechanisms, offering a safer alternative in relation to conventional chemotherapy drugs such as Docetaxel, Brachydin B ability to promote cytotoxicity in 2D and 3D models further reinforces its therapeutic promise. Together, these studies encourage additional exploration of Brachydins as viable options for the treatment of metastatic prostate cancer, possibly with lower toxicity for normal cells.^21,24^

The antiproliferative and cytotoxic potential of the crude hydroethanolic extract and its subfraction, which contains 59.3% of Brachydin E and 40.7% of Brachydin F, as well as two composts isolated from the roots of *F. platyphylla* foram investigated. The crude hydroethanolic extract, subfractionated and the Brachydin E and Brachydin F composts obtained according to Rocha et al., Por meio liquido–liquido partição com CH_2_Cl_2_ e H_2_O-MeOH (7:3). The hydromethanolic fraction was fractionated by column chromatography, resulting in a subfraction containing Brachydin E and F, whose quantity was determined by HPLC-PDA and analyzed by NMR to confirm purity. Cytotoxic activity was assessed using the MTT method in cell lymphocytes from glioblastoma (U-251), lung (NCI-H460), prostate (PC-3) and colon (HT-29). The data indicate that the isolated extracts and composts of *F. platyphylla* present variable cytotoxic activities according to the form of the extract and the cell lines tested. The crude hydroethanolic extract had an IC_50_ of 95.8 μg/mL in U-251 (glioblastoma) and did not show significant effects in other cases, suggesting the presence of components that could dilute its effectiveness. In contrast, a subfraction, enriched in dimeric flavonoids Brachydin E and F, exhibited better performance, with IC_50_ of 47.7 μg/mL in U-251 and 42.5 μg/mL in PC-3 (prostate). Noteworthy is Brachydin E, which presented an IC_50_ of 5.9 μg/mL in PC-3, indicating promising therapeutic potential. Comparing doxorubicin as a positive control, which has an IC_50_ of 2.1 μg/mL in PC-3, the flavonoids show antitumor efficacy, but with lower potencies. The IC_50_ values for Brachydin F foram reported as 33.1 μg/mL in PC-3 and 44.1 μg/mL in U-251, indicating that, although less potent than Brachydin E, it also presents significant cytotoxic activity. The selectivity of two composts, especially Brachydin E, against tumor cells in relation to healthy cells, suggests potential therapeutic use in oncological treatments, deserving more in-depth investigation.^9^

### 3.5 Potential Antimicrobial Agent

Studies with *F. platyphylla* demonstrate its antifungal potential against several strains of Candida, including *C. tropicalis*, *C. guilliermondii*, *C. albicans*, and *C. krusei*, with the hydroethanolic extract showing inhibitory activity.^31^ The flower extract was obtained by maceration method with a 70% hydroethanolic solution. The antifungal activity on Candida sp. was carried out using paper disk diffusion methodology, and the cytotoxic activity on *A. salina*, both assays in different concentrations of extract. The results will show that the *F. platyphylla* floral extract presents significant inhibitory activity against *C. guilliermondii*, with inhibition zones varying from 13.8 to 9.7 mm and against *C. albicans*, with measurements of 18.6 to 14.5 mm in higher concentrations of 50 to 100 mg/mL, respectively. Furthermore, the cytotoxicity of extracts of *F. platyphylla* was evaluated in tests with *A. salina*. The extracts demonstrate toxicity, with Letal concentration (LC_50_) values of 237.81 μg/mL, which highlights its potential as a source of antifungal and cytotoxic agents.^25^ Unfortunately, minimum inhibitory concentrations (MIC) and chemical composition were not determined.

The essential oil in the flower also inhibits phytopathogenic fungi such as *S. sclerotiorum* and has antioxidant activity. *Brachydin-B* demonstrated antifungal activity against *C. albicans*, and the hydroethanolic extract and the dichloromethane fraction increased the efficacy of the antibiotic Norfloxacin against *S. aureus*, suggesting a potential to alter microbial resistance. These results highlight *F. platyphylla* as a promising source of antifungal compounds, indicating the need for further studies on its mechanisms of action and therapeutic potential.^22^

### 3.6 Microemulsion is a Potential Delivery System for Enhanced Therapeutic Efficacy and Safety

The research by Nascimento et al. showed that microemulsion developed, incorporating a flavonoid-rich compound from the roots of *F. platyphylla*, releases the active compound more efficiently than soluble forms and presents excellent physical and chemical stability. The fraction dichloromethane (DCM) extracted from the roots of *F. platyphylla* is rich in dimeric flavonoids, specifically the composts made as brachydins A, B and C. The development of microemulsion used a pseudoternary diagram to identify the ideal proportions of water, isopropyl myristate and surfactants, resulting in white microemulsifiers (ME), with 3% (ME3) and 5% (ME5) of the DCM fraction. ME3 has particle sizes less than 100 nm, stability under extreme conditions and favorable organoleptic characteristics, such as transparency, physiologically oily pH, refractive index of 1.42 and density of 1.017 g/cm^3^. Stability tests indicate that microemulsifiers do well even after exposure to extreme thermal conditions, with minimal variations in pH and the concentration of the incorporated fraction. The *in vitro* release study showed that ME3 provided a controlled release of the fraction, with more than 60% released in 6 h. Additionally, toxicity tests of the DCM fraction and the ME3 microemuls were carried out using *Tenebrio molitor* larvae as an experimental model. The larvae, saudaveis and weighing between 100 and 200 mg, were distributed in 14 groups, with injections of 10 μL of substances in concentrations of 1 to 100 μg/mL. Control groups were used to ensure precise results. The viability was monitored for 7 days, and the results will show that both substances do not cause toxicity, indicating a safety profile suitable for potential therapeutic applications. The toxicity assessment in *T. molitor* larvae confirmed the safety of both the microemulsion and the flavonoid-rich DCM fraction, with no evidence of toxicity at the levels tested. These results indicate that microemulsions can improve the efficacy of *F. platyphylla* extracts, enhancing their therapeutic effects and ensuring safety and stability. In this sense, the integration of microemulsions in the formulation of herbal medicines can represent a significant advance for the clinical use of *F. platyphylla* compounds.^32^

### 3.7 Potential Pharmacological Agents: Benefits and Safety Considerations

Studies on *F. platyphylla* reveal several potential pharmacological uses of the extract and essential oil.^33^ However, as mentioned above, it is crucial to consider the possible mutagenic effects of plant extracts.^33,34^ Research by Resende et al. indicated that all extracts and the aqueous fraction of *F. platyphylla* leaves were mutagenic, with the crude extract showing better activity. Furthermore, only the hydroalcoholic extract of the roots showed significant estrogenic activity.^35^ Although it was noted many pharmacological benefits, most studies were *in vitro*, which highlights the need for further *in vivo* investigations to deepen the understanding of the plant’s mechanisms of action.

### 3.8 Therapeutic and Nanotechnological Prospects of Bioactive Compounds from *F. platyphylla*: Toward Clinical Application

Among the bioactive constituents derived from *F. platyphylla*, brachydins (notably A, B, C, E, and F) have emerged as key pharmacophores, exhibiting a wide range of therapeutic activities linked to their unique dimeric flavonoid structures. These dimeric flavonoids have been implicated in various biological activities, including antiparasitic, antitumor, antifungal, anti-inflammatory, antioxidant, and antinociceptive effects, highlighting their potential as selective antitumor agents.^8,9,11,28,36^ Luteolin, a well-studied flavonoid, exhibits significant antiproliferative effects on glioblastoma cells with minimal toxicity to nontumor cells, reinforcing its relevance as a therapeutic candidate. Additionally, conandroside has emerged as a potential inhibitor of lipoxygenase, an enzyme involved in the inflammatory cascade, thereby positioning this compound as a potential anti-inflammatory agent.^5^

Halogenated compounds derived from this species have also demonstrated crucial antiparasitic activity against *L. amazonensis* and *T. cruzi*, presenting high selectivity. These findings underscore the feasibility of developing new antiparasitic drugs based on these compounds. Also, *F. platyphylla* exhibits promising antifungal and antibacterial potential, with extracts inhibiting *Candida spp*. and bacteria such as *S. aureus*. Compounds like brachydin B show antifungal activity, and the leeves extracts enhance the efficacy of antibiotics such as norfloxacin, suggesting a modulatory effect on microbial resistance.^17^ These findings support its potential use in antimicrobial therapies and highlight the need for further studies investigating mechanisms of action, safety, and clinical efficacy.

In parallel, nanotechnology-based systems have proven to be highly effective strategies for improving the bioavailability of active compounds, regulating their release profiles, enhancing solubility, and minimizing systemic toxicity.^4,10,37^ The application of advanced drug delivery systems, such as microemulsions, has further strengthened the therapeutic prospects of *F. platyphylla*.^32^ These attributes are critical to optimizing the pharmacokinetic and pharmacodynamic profiles of phytocompounds in translational medicine.

In the technological context, the microemulsion formulated with dimeric flavonoids isolated from *F. platyphylla* exhibits key nanopharmaceutical attributes, including nanometric droplet size (ME3:65.4  ±  9.8 nm), physicochemical stability, and sustained drug release (>60% within 6 h). Although the formulation presented a moderate polydispersity index (PDI = 0.543 ±  0.11), its favorable ζ-potential and near-physiological pH reinforce its potential for biomedical use. Importantly, *in vivo* toxicity assays using *Tenebrio molitor* larvae confirmed the safety of both the dichloromethane extract and ME3, supporting its viability as a nanocarrier system for bioactive phytocompounds.^32^

The encapsulation of brachydin A in F127 micelles—an FDA-approved copolymer—resulted in high encapsulation efficiency (92.65 ±  0.48%) and favorable nanometric profiles, with particle sizes ranging from 157 to 359 nm and low polydispersity indices. These characteristics ensure pharmacokinetic stability and enhance bioavailability. Notably, the worm-like micellar architecture may prolong plasma half-life and reduce macrophage uptake, contributing to selective leishmanicidal activity with minimal cytotoxicity. Collectively, these findings highlight the potential of nanostructured systems to enhance brachydin A delivery and therapeutic precision.^21^

In parallel with previous strategies, zein nanoparticles encapsulating the dichloromethane fraction (DCMF) of *F. platyphylla*, rich in brachydins A, B, and C, demonstrated excellent nanotechnological performance, with high encapsulation efficiency (99.8%), narrow size distribution (mean diameter ∼ 206 nm), and structural stability. This formulation significantly enhanced the antiparasitic activity of the bioactive fraction, reducing IC_50_ values from 253.1 to 36.33 μg/mL (promastigotes) and from 6.96 to 0.72 μg/mL (amastigotes) of *L. amazonensis*. Moreover, cytotoxicity to RAW 264.7 macrophages remained minimal (CC_50_ > 500 μg/mL), yielding a high selectivity index (SI = 694.4) for the intracellular form. These findings suggest that zein-based nanocarriers promote improved bioavailability, membrane permeability, and targeted delivery of flavonoid compounds, offering a promising platform for the development of plant-derived antileishmanial therapies.^27^

Although preliminary *in vitro* and *in vivo* findings are encouraging, comprehensive preclinical studies remain essential to ensure these compounds’ safe and effective translation into clinical use.^38,39^ In particular, acute and subchronic toxicity assessments and pharmacokinetic and pharmacodynamic analyses in animal models are critical for understanding how these substances behave in a complex biological system.^40^ *In vivo* studies are indispensable because they provide insights into absorption, distribution, metabolism, and excretion (ADME) and potential systemic effects and organ-specific toxicity—factors that cannot be fully predicted by *in vitro* assays alone.^39,40^ These evaluations form the scientific basis for defining safe dose ranges and identifying potential risks.^40^ Following this stage, phase I clinical trials are required to confirm human safety, tolerability, and preliminary pharmacological responses, serving as the gateway to further therapeutic development.^41^

In addition, future research should prioritize several key areas to support the translational development of *F. platyphylla* compounds. One critical focus is the investigation of pharmacological synergism,^42^ particularly the interaction between brachydins and luteolin, as well as their combined application with conventional chemotherapeutic agents. These combinations should be evaluated using tools such as the Combination Index (CI), which can quantify synergistic effects and potentially enable effective dose reduction while minimizing toxicity.^42,43^

Equally important is the standardization of extracts and their isolated compounds, which includes the quantification of key bioactive markers, such as brachydins and luteolin, through chromatographic techniques. This process should be complemented by formulation studies that evaluate the stability, solubility, and viability of various dosage forms, including tinctures, capsules, and microemulsions, which may improve pharmacokinetic profiles and patient adherence.^44^

To ensure translational relevance, clinical validation through Phase I trials is essential. These studies will provide fundamental data on human safety, tolerability, and initial pharmacodynamics, forming the basis for subsequent clinical development.^45,46^ Furthermore, assessing the technological scalability and pharmaceutical viability of these compounds, through feasibility studies on large-scale extraction, formulation, and production, will be vital for their incorporation into therapeutic protocols.^46,47^

While *F*. platyphylla and its isolated compounds—particularly brachydin B—have demonstrated promising pharmacological activities in preclinical models, including selective antitumor, antiparasitic, and anti-inflammatory effects, their potential clinical application must be interpreted with caution. The absence of clinical trials, combined with reports of mutagenic and estrogenic activity in some extracts, highlights the urgent need for comprehensive safety assessments. Until robust toxicological and pharmacokinetic data are available, the therapeutic use of these compounds should be considered exploratory and restricted to experimental investigation.

## 4 Conclusions

This integrative review provides, for the first time, a consolidated and critical overview of the phytochemical diversity, pharmacological properties, and toxicological data available on *F. platyphylla*. By synthesizing fragmented findings from 20 original studies across both *in vitro* and *in vivo* models, this work highlights the scientific relevance of the species as a promising yet underexplored platform for natural product-based drug discovery. The systematic presentation of data according to plant parts, compound classes, and experimental models underscores the novelty and utility of this compilation, which fills a significant gap in the literature.

Among the bioactive constituents, the dimeric flavonoids known as brachydins (A–F) emerged as the most pharmacologically potent, particularly brachydin B, which demonstrated selective antitumor and antimetastatic effects in 2D and 3D prostate cancer models. Novel compounds such as brachydins E and F, identified for the first time in this species, exhibited selective cytotoxicity against tumor cells. Luteolin, another relevant compound, exhibited antiproliferative effects on glioblastoma cells with minimal toxicity to nontumor cells, further supporting the therapeutic potential of this phytochemical group.

Technologically, the development of a microemulsion incorporating a dichloromethane root extract resulted in a stable delivery system with controlled release and favorable *in vivo* tolerability, thereby reinforcing the potential for translational pharmaceutical applications.

Nevertheless, the clinical applicability of these findings remains hypothetical and should be approached with caution. Reports of mutagenic and estrogenic activity in some extracts, particularly from leaves and roots, raise significant safety concerns. Moreover, the lack of clinical trials and the predominance of preliminary *in vitro* data limit the immediate translational relevance of these compounds. Thus, while the pharmacological evidence is encouraging, no therapeutic recommendation can be made at this stage.

To bridge this gap, future investigations should prioritize the characterization of pharmacokinetic behavior, bioavailability, dose–response relationships, and long-term toxicity of both crude extracts and isolated constituents. Robust *in vivo* validation, mechanistic elucidation, and standardized preclinical protocols will be essential to support the rational and safe development of *F. platyphylla*-derived bioactive agents for potential clinical use.

## 5 Materials and Methods

### 5.1 Integrative Review Strategies

A scientific review was conducted to probe relevant studies through an integrative literature search in the PubMed, Scielo, and Google Scholar databases. The search was limited to articles written in the last ten years, between October 2014 and December 2024. The search included keywords such as “*F. platyphylla* AND *in vivo* studies”, “*F. platyphylla* AND *in vitro* studies”, “*F. platyphylla* AND ethnopharmacological studies”, “*A. brachypoda* AND *in vivo* studies”, “*A. brachypoda* AND *in vitro* studies”, and “*A. brachypoda* AND ethnopharmacological studies” in English. Additionally, the reference lists of the retrieved studies were scanned to identify any potentially missed articles.

After searching the databases using the keywords, 896 studies addressing the theme of this review were identified (Table 4). Of this total, the results associated with the keyword *A. brachypoda* obtained the most published articles. Furthermore, the Google Scholar database yielded the most available articles (874), followed by PubMed (20) and Scielo (2).

**Table 4:** Search of Databases for Scientific Articles Published between October 2014 and December 2024 with the Species *F. platyphylla* or Its Synonym *A. brachypoda* ^a^

keywords	PubMed	Scielo	Scholar Google
*A. brachypoda*	10	01	363
*A. brachypoda* AND *in vivo* studies	02	00	74
*A. brachypoda* AND *in vitro* studies	00	00	117
*A. brachypoda* AND ethnopharmacological studies	00	00	24
*F. platyphylla*	07	01	221
*F. platyphylla* AND *in vitro* studies	01	00	27
*F. platyphylla* AND *in vivo* studies	00	00	20
*F. platyphylla* AND ethnopharmacological studies	00	00	28
**Total**: 896	20	02	874

a Prepared by the authors from the PubMed, Scielo, and Google Scholar databases.

### 5.2 Study Selection Criteria

After conducting the searches, the records were imported into EndNote software. Any duplicate articles were then removed. Two researchers independently reviewed the titles and abstracts of all citations to select only the relevant studies.

In the characterization aspects of the included studies, several criteria were adopted, such as the type of study, study objective, administration of the formulation, and treatment effects. Then, all the necessary data were organized and presented in tabular form. Data not identified in the studies were filled with n.a (not evaluated). An overview of the excluded and included studies is presented in Figure 7, prepared under the Preferred Reporting Items for Systematic reviews and Meta-Analyses statement (PRISMA).^48^ Also, two investigators independently determined whether the studies met the inclusion criteria, with a third resolving any disputes as necessary.

**Figure 7:**
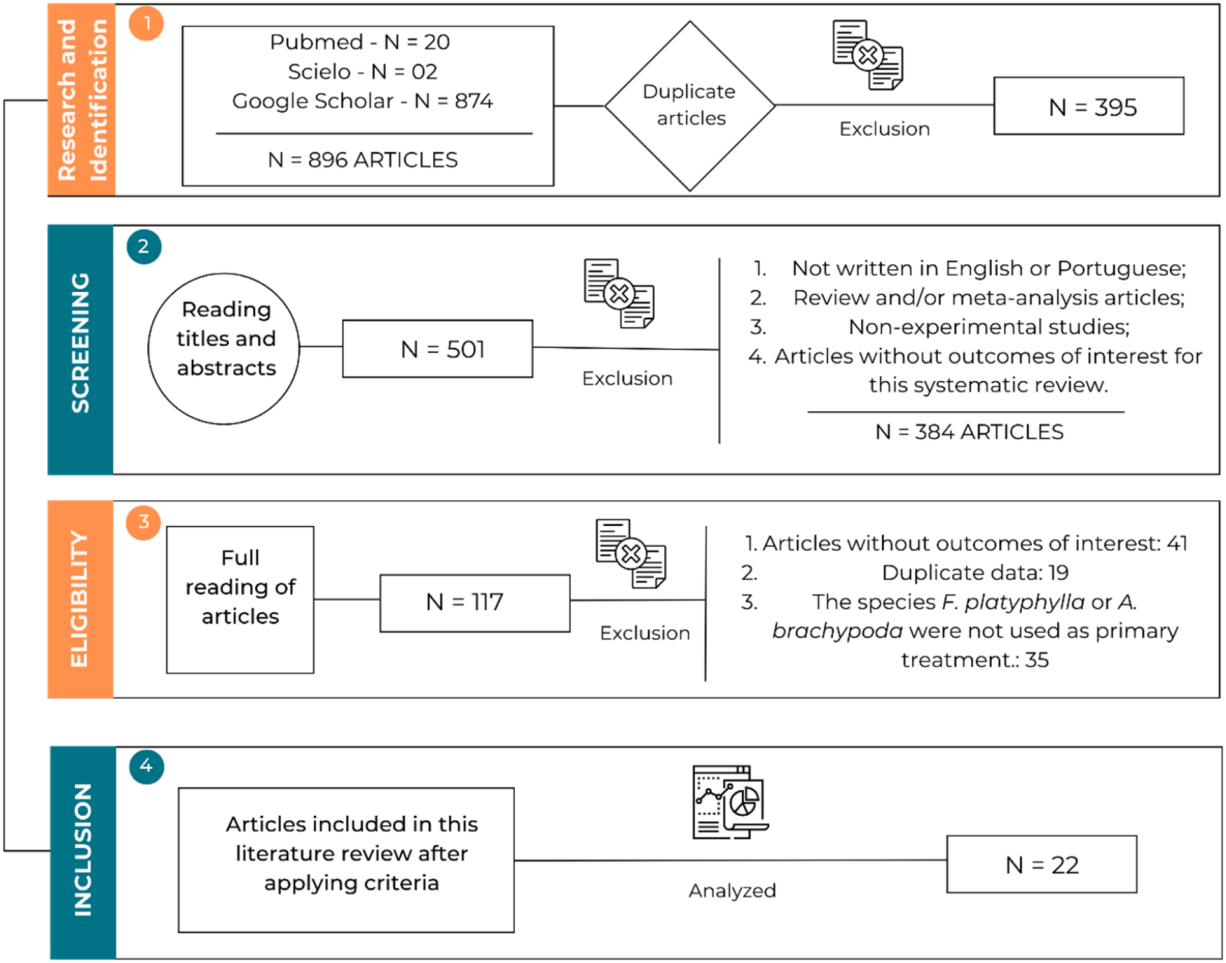
Flowchart of the search strategy and study selection **Source**: Prepared by the authors based on the studies included in this review and prepared under the PRISM 2020.

Of the 896 records initially identified through database searches, 395 were excluded as duplicates. The remaining 501 records were screened based on title and abstract, resulting in the exclusion of 479 articles that did not meet the eligibility criteria—namely: not written in English or Portuguese, classified as reviews or meta-analyses, lacking experimental data, not reporting relevant outcomes for this review, or not using *F. platyphylla* (or its synonym *A. brachypoda*) or their constituents as the primary focus of investigation. After full-text assessment, 22 studies met all inclusion criteria and were retained for detailed qualitative analysis in this review.

### 5.3 Data Extraction, Methodology, and Findings

A data extraction form was developed in Microsoft Excel and included information regarding study design, group characteristics, pharmaceutical formulation identification, and main findings (Tables 2, 3, and 4). Data extraction was performed independently by two reviewers and compared for disparities.

### 5.4 Data Synthesis and Analysis

A narrative synthesis was performed, categorizing the studies according to their characteristics and settings. The effects of the pharmaceutical formulation were inferred using experimental trials. The researchers chose not to conduct a meta-analysis because the included studies varied in aspects such as study designs, participant groups, pathologies evaluated, variable definitions, comparisons, and analytical strategies. The structure of this methodology was taken as an example from the one recommended by Donato and Donato.^49^
